# An antibiotic derivative as a new potential tool in the prevention of hemolytic uremic syndrome

**DOI:** 10.1016/j.isci.2025.113076

**Published:** 2025-07-07

**Authors:** Elisa Varrone, Luciano Consagra, Domenica Carnicelli, Elisabetta Galassi, Beatrice Munari, Elisa Porcellini, Marta Pluchino, Giorgia Rossi, Federico Parenti, Catia Barboni, Barbara Brunetti, Francesca Ricci, Pier Luigi Tazzari, Francesco Manoli, Ilse Manet, Paola Paterini, Gianluca Storci, Massimiliano Bonafè, Alejandro Hochkoeppler, Anna Zaghini, Stefano Morabito, Gianluigi Ardissino, Timo Vaara, Martti Vaara, Maurizio Brigotti

**Affiliations:** 1 Department of Medical and Surgical Sciences (DIMEC), University of Bologna, 40126 Bologna, Italy; 2 Department of Veterinary Medical Sciences (DIMEVET), University of Bologna, Ozzano dell'Emilia, 40064 Bologna, Italy; 3 IRCCS Azienda Ospedaliero-Universitaria di Bologna, 40138 Bologna, Italy; 4 Istituto per la Sintesi Organica e la Fotoreattività, Consiglio Nazionale delle Ricerche, 40129 Bologna, Italy; 5 Center for Applied Biomedical Research-CRBA, University of Bologna, IRCCS Azienda Ospedaliero-Universitaria di Bologna, 40138 Bologna, Italy; 6 Department of Pharmacy and Biotechnology, University of Bologna, 40136 Bologna, Italy; 7 European Reference Laboratory for Escherichia coli, Istituto Superiore di Sanità, 00161 Rome, Italy; 8 Center for HUS Control, Prevention and Management, Fondazione IRCCS Ca’ Granda Ospedale Maggiore Policlinico, 20122 Milan, Italy; 9 Northern Antibiotics Ltd, 00140 Helsinki, Finland; 10 Reconstructive Orthopaedic Surgery and Innovative Techniques – Musculoskeletal Tissue Bank, IRCCS Istituto Ortopedico Rizzoli, 40136 Bologna, Italy

**Keywords:** Pharmacology, Natural sciences, Biological sciences, Microbiology, Medical Microbiology

## Abstract

Hemolytic uremic syndrome (HUS), the main cause of acute renal failure in early childhood, is associated with infections by *Escherichia coli* strains producing Shiga toxin 2 (Stx2). The microangiopathic injuries caused by the toxin occur mainly in the renal microvasculature when the glycolipid receptor globotriaosylceramide (Gb3Cer) is targeted. Before entering the kidney, Stx2 binds to circulating cells through Gb3Cer and Toll-like receptor 4 (TLR4) and is subsequently delivered in extracellular vesicles to target cells. Here, we have found a specific inhibitor of the Stx2/TLR4 interaction, the preclinical polymyxin B derivative NAB815. The compound impairs the formation of Stx2-containing extracellular vesicles produced by leukocytes and platelets and also reduces their toxic effects in cellular (Vero cells) and animal models (CD-1 mice). NAB815 would represent a useful tool in preventing HUS and is effective at sub-bactericidal concentrations, thus overcoming the concern that antibiotics are harmful to patients infected with Stx2-producing *E. coli*.

## Highlights

• In children, life-threatening HUS is caused by bacteria producing Shiga toxins
• There are no specific treatments for HUS, and antibiotics are not recommended
• We have found an antibiotic impairing toxin actions at sub-bactericidal concentration
• Administration of this drug in patients may represent a useful tool in preventing HUS

## Graphical abstract

**Figure:**
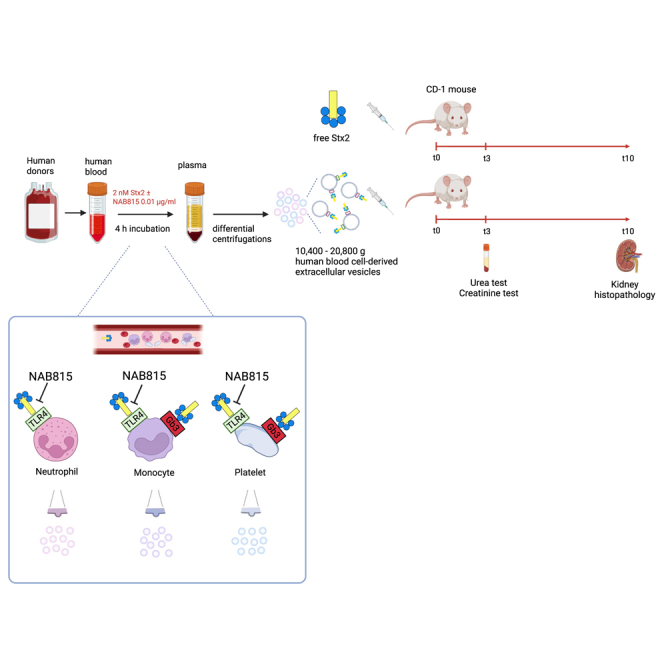

## Introduction

Shiga toxins (Stx) are bacterial proteins consisting of a pentamer of B subunits that are non-covalently bound to a single A subunit, which confers the enzymatic activity responsible for toxicity.^1^ The two Stx types named Stx1 and Stx2 produced by certain pathogenic *Escherichia coli* strains called Shiga toxin-producing *E. coli* (STEC) are the crucial virulence factors for the development of hemolytic uremic syndrome (HUS).^2^^,^^3^^,^^4^ HUS is a severe consequence of these food-borne or environmentally related intestinal infections and affects approximately 10%–15% of STEC-infected patients, especially children younger than 3 years.^2^ These infections are considered a global public health issue.^5^^,^^6^^,^^7^ According to a recent WHO report,^8^ between 1998 and 2017, 957 STEC outbreaks were described in 27 countries. HUS can manifest even in the form of isolated cases or small clusters.^2^

HUS is characterized by the triad hemolytic anemia, thrombocytopenia, and acute renal failure, which manifest approximately 1 week after the beginning of the prodromal intestinal phase (first loose stool) when Stx have already gained access to the circulation. Indeed, the toxins are produced by the bacteria in the intestine and are subsequently released into the bloodstream where they interact with human circulating cells (early toxemia) before intoxicating the endothelia of the kidneys and brain, as well as other renal cells, triggering HUS (late toxemia).^2^^,^^3^^,^^4^ The target cells express the glycolipid receptor globotriaosylceramide (Gb3Cer), which interacts with the pentamer of B subunits of Stx.^9^ The interaction with circulating cells during early toxemia plays a crucial role in the pathogenesis of HUS and can take place through Gb3Cer expressed on human monocytes and platelets.^10^^,^^11^ In 2013, we identified another cellular receptor involved in the interaction between Stx and human circulating cells (neutrophils, monocytes, and platelets) known as Toll-like receptor 4 (TLR4), which interacts with the A subunit of Stx.^12^^,^^13^

Soluble factors, such as human serum amyloid P component (HuSAP) present in human blood, could affect the binding and toxicity of Stx2. This circulating factor, composed of a pair of pentameric subunits, is a negative modulating factor of Stx2. Each subunit binds specifically to Stx2, and not to Stx1, impairing its interaction with Gb3Cer receptors and thus inhibiting its cytotoxic activity.^14^^,^^15^^,^^16^ At the same time, HuSAP stimulates the binding of Stx2 to TLR4 expressed by human neutrophils.^17^

The striking features observed in the blood of patients affected by HUS were the formation of leukocytes/platelet aggregates and large extracellular vesicles (EVs) containing Stx and other virulence factors involved in the development of the syndrome.^18^^,^^19^^,^^20^ The mechanism of formation of both aggregates and EVs relies on the multiple interactions of Stx with monocytes, neutrophils, and platelets through the two aforementioned receptors (Gb3Cer and TLR4).^3^ Monocytes and platelets bear both receptors, whereas human neutrophils lack Gb3Cer^21^ and only interact with Stx through TLR4.^13^ The circulating cells are activated after toxin binding and, eventually, form aggregates and EVs. EV-associated Stx damage renal endothelial cells and other renal cells, as well as brain endothelium, and this triggers the formation of microthrombi that reduce renal flow, consume platelets, and damage passing erythrocytes causing the HUS triad.^3^ In the kidneys of patients with HUS, the occurrence of 1-μm-diameter EVs containing Stx2a was demonstrated by electron microscopy,^22^ hence confirming the pivotal role of these structures.

Stx2a is the toxin subtype most frequently related to the development of HUS.^23^ Notably, Stx2a can be hosted inside EVs and can also be exposed on the external side of the EV membrane. When the exposed Stx2a is bound to TLR4 through its A chain, the B chain pentamer is free to associate with target endothelial cells that express Gb3Cer, and, hence, the Stx2a contained within the vesicle, and other virulence factors, is delivered.^3^^,^^20^ This ominous form of circulating toxin (Stx2a bound to EVs through A chain) appears in the blood of STEC-infected patients the day before the development of HUS, whereas it is absent in patients who do not progress to HUS.^20^

There are no specific therapeutic interventions for HUS: patients are treated with supportive therapy (replacement of fluids and electrolytes, hyperhydration, dialysis, and blood transfusions).^4^^,^^24^^,^^25^ Despite the infectious origin of typical HUS, antibiotics are not recommended for the treatment of STEC infections since their use has been shown to increase the risk of HUS, likely due to increased Stx expression and release in patients’ blood.^4^^,^^26^^,^^27^

**Figure 1:**
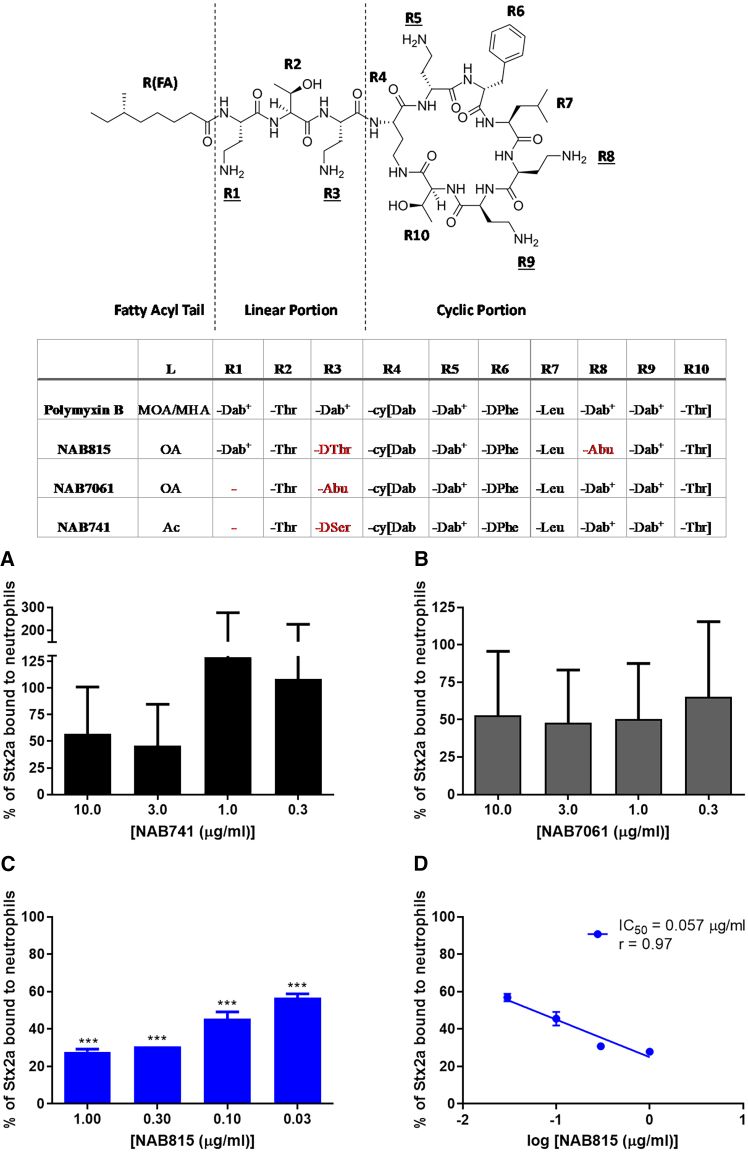
Effect of the polymyxin B derivatives NAB741, NAB7061, and NAB815 on the binding of Stx2a to human neutrophils Isolated neutrophils (5 × 10^5^/mL) were incubated 90 min at 37°C with Stx2a (60 nM) in 250 μL of PBS-BSA 1% in the presence or in the absence of different concentrations of the polymyxin B derivatives NAB741, NAB7061, and NAB815 (resuspended in PBS at the concentration of 10 mg/mL and diluted in the same buffer). After incubation, cells were sedimented by centrifugation at 200*g* for 5 min and washed three times with 100 μL of the same buffer at 37°C. The binding of Stx2a was assessed by indirect flow cytometric analysis as described in STAR Methods. (A–C) Percentage of binding obtained with neutrophils from different human donors (mean ± SD; *n* = 3) relative to the value obtained in the presence of toxin alone (mean channel value of fluorescence = 2.4 ± 0.3, for A and B; 4.3 ± 0.9, for C). (D) Data in (C) were plotted to calculate the IC_50_ of NAB815 in inhibiting the binding of Stx2a to human neutrophils. The IC_50_ was calculated by the linear regression between the mean percentage of neutrophil-bound Stx2a and the log of NAB815 concentrations. ∗∗∗p < 0.001 (two-tailed unpaired t test) relative to the sample with toxin alone. The Pearson correlation coefficient (*r*) was used to assess the correlation between variables. Inset: structure of polymyxin B showing the linear (R1–R3) and cyclic (R4–R10) portions of the antibiotic, and the lipidic tail, R(FA). The underlined residues (R1, R3, R5, R8, and R9) bear a positive charge. Reproduced with permission from Molecules 24, 249 (2019). The substitutions of specific amino acids in the polymyxin B derivatives are also marked in red. Dab, 2,4-diaminobutyric acid; Abu, 2-aminobutyric acid; Thr, L-threonine; DThr, D-threonine; DPhe, D-phenylalanine; Leu, leucine; DSer, D-serine; OA, octanoyl acid; MOA, methyloctanoic acid; MHA, methylheptanoic acid; Ac, acyl; and cy[…], a cycle consisting of the components indicated in the square brackets and in which the first and the last components are bound. See also Figures S1, S3, and S4.

Polymyxin B is an antibiotic active against gram-negative bacteria,^28^ and it is known to block the interaction between the bacterial endotoxin (lipopolysaccharide [LPS]) and TLR4.^29^^,^^30^^,^^31^ In 2016, we showed that polymyxin B effectively impairs the Stx2a/TLR4 interactions.^32^ However, polymyxin B is nephrotoxic and neurotoxic.^33^ Furthermore, it does block the binding of Stx2a to leukocytes at μg/mL concentrations that are also highly bactericidal. Polymyxin B is a pentacationic cyclic lipodecapeptide (Figure 1, inset) bearing three positive charges in the cyclic portion, spanning from the residues R4 to R10, and two positive charges in the linear portion formed by residues R1 to R3 (underlined residues in Figure 1, inset). To possibly reduce the nephrotoxicity, we designed polymyxin B derivatives with fewer cationic charges.^28^^,^^33^^,^^34^ NAB741 and NAB7061 are shorter polymyxin B derivatives (lipononapeptides) with three positive charges in the cyclic portion due to the deletion of Dab^+^ at R1 and substitution of DSer (NAB741) or Abu (NAB7061) for Dab^+^ at R3 in the linear portion^35^^,^^36^ (Figure 1). The most recent derivative is NAB815, a lipodecapeptide in which the reduction of positive charges from five to three was obtained by the substitution of DThr for Dab^+^ at R3 in the linear portion and Abu for Dab^+^ at R8 in the cyclic portion (Figure 1, inset).^37^ NAB815 has a cytotoxicity to human renal tubular cells (IC_50_, 334 μg/mL) approximately 20 times lower than that of polymyxin B (IC_50_, 18 μg/mL).^37^ Furthermore, in a 7-day cynomolgus monkey study, NAB815 at 36 mg/kg/day (divided into three daily doses) was well tolerated. There was no significant increase in serum creatinine, blood urea nitrogen, or in the urinary biomarkers tested. Contrarily, all animals receiving polymyxin B at 18 mg/kg/day had to be euthanized because of severe nephrotoxicity.^38^ The derivative is still at the preclinical stage of development but shows promise in the treatment of complicated urinary tract infections. In the murine *E. coli* pyelonephritis model, it is 10 times more efficient than polymyxin B,^39^ due to the fact that, compared to polymyxin B, its excretion into urine is approximately 10 times more efficient.^40^

Herein, NAB815 was found to be effective at sub-bactericidal concentrations in blocking the interactions of Stx2a with human circulating cells and preventing the functional consequences related to HUS pathogenesis, i.e., the formation of white cell/platelet aggregates and the release of Stx2-containing EVs.

## Results

### NAB815 is a good inhibitor of the interaction between Stx2a and TLR4

The binding of Stx2a to TLR4-expressing Gb3Cer-lacking human neutrophils was measured by indirect cytofluorimetric analysis after incubation of cells from three donors with the toxin (Figure 1). In particular, the effects induced by the addition of different derivatives of polymyxin B were compared by setting at 100% the fluorescence values obtained in the absence of the putative inhibitors that corresponded to the maximal amount of Stx2a bound to neutrophils (Figures 1A–1D). Both NAB741 and NAB7061 proved to be scarcely effective as inhibitors of the binding of Stx2a to neutrophils since they did not induce significant dose-dependent effects (Figures 1A and 1B). It should be noted that NAB7061 and NAB741 are capable of perturbing the outer bacterial membrane^33^; therefore, non-specific effects on the cell membrane could be envisaged. In contrast, NAB815 strongly inhibited neutrophil/Stx2a interactions, providing reproducible results with marked dose-dependent effects even at concentrations below 1 μg/mL (Figures 1C and 1D). The IC_50_ (NAB815 concentration causing 50% inhibition of toxin binding to neutrophils) calculated by the curve shown in Figure 1D (0.057 μg/mL) is approximately 60 times lower than that obtained with the parental antibiotic polymyxin B (3.5 μg/mL) under similar conditions.^32^ It should be noted that the binding of Stx2a to neutrophils is obtained by incubating cells with 60 nM toxin and is inhibited by 50% in the presence of 43 nM NAB815 (0.057 μg/mL), which suggests a direct Stx2a/NAB815 interaction at a 1:1 stoichiometric ratio.

Gb3Cer-expressing cells, such as human Raji cells and simian Vero cells,^41^^,^^42^ treated with Stx2a are not protected by NAB815 in a cell-translation assay^43^ (Figure S1), as previously shown with polymyxin B.^32^ To sum up, NAB815 specifically inhibits TLR4/Stx2a interactions.

### NAB815 binds directly to Stx2a

**Figure 2:**
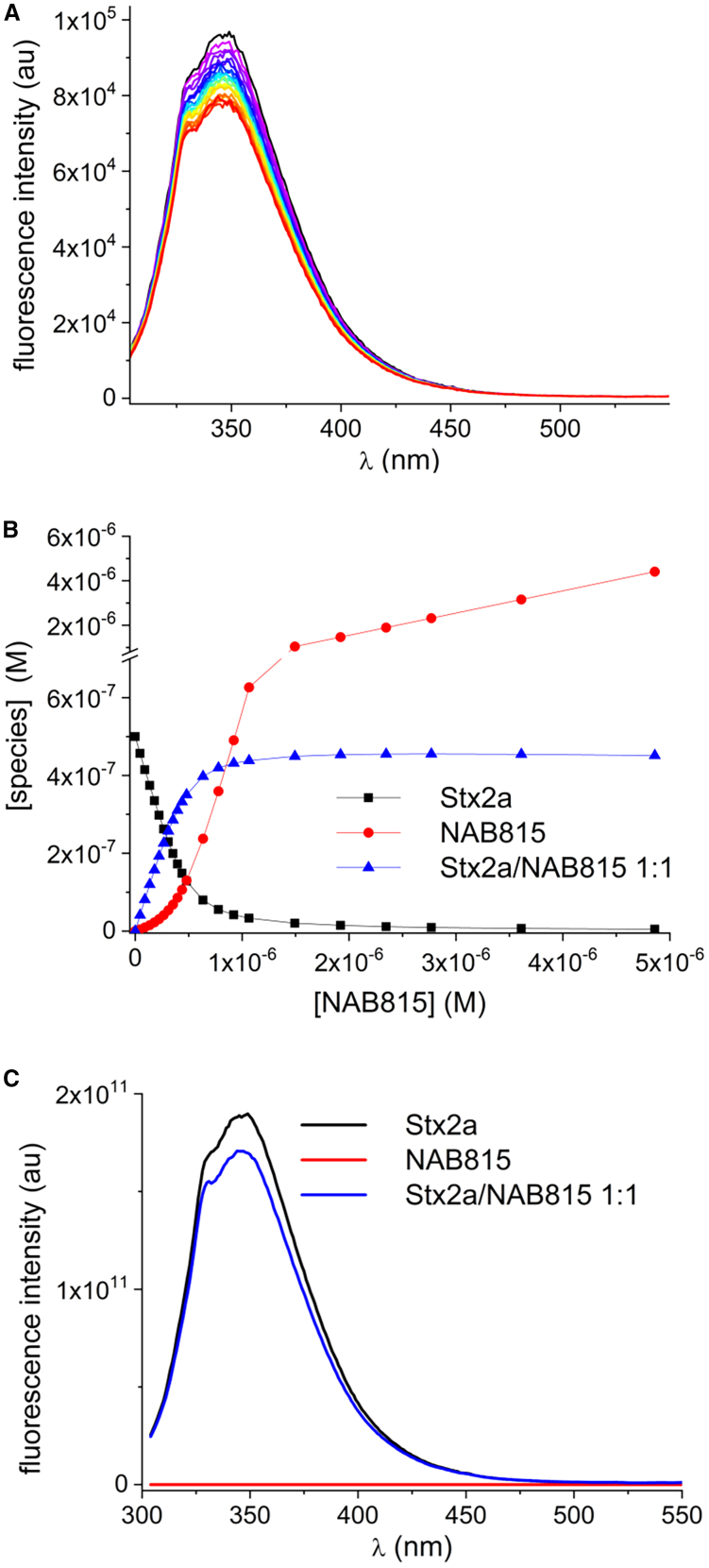
Determination of the binding of NAB815 to Stx2a Stx2a (0.5 μM in 300 μL of PBS) was excited at 295 nm to collect the fluorescence spectrum with a maximum value at 349 nm. Subsequently, increasing amounts of NAB815 were added to the final concentrations of 0.05–5 μM, and the fluorescence was measured after each addition (volume of the sample at the end of the assay, 326 μL). The results were corrected at each experimental point for the dilution of the toxin due to the addition of the antibiotic. NAB815 excited at the same wavelength is not fluorescent. (A) Fluorescence spectra of the titration of Stx2a (black line) with increasing amounts of NAB815 (colored lines). (B) Plot of the equilibrium concentrations of the various species (Stx2a, NAB815, and the 1:1 complex) vs*.* the total concentration of NAB815. (C) Calculated fluorescence spectra of Stx2a as free species and complexed with NAB815. See also Figure S2.

To give direct evidence of the NAB815/Stx2a interaction, we exploited the natural intrinsic fluorescence of Stx2a due to the 12 tryptophan residues present in the molecule. By exciting the toxin at 295 nm, we have obtained a fluorescence spectrum (maximum at 349 nm) that was progressively quenched by increasing amounts of NAB815 until reaching a plateau corresponding to a maximum reduction by approximately 10% (Figure 2A). The quenching may be due to the presence of amine functions in NAB815 and involve electron transfer from the amine to tryptophan. Analysis of the spectra converged by applying a model with a 1:1 NAB815/Stx2a complex and afforded the complex dissociation constant (K_d_ = 5 × 10^−8^ M), indicating a good affinity of NAB815 for Stx2a. The species concentrations at equilibrium and the calculated spectra of free and complexed Stx2a are shown (Figures 2B and 2C). The 10% maximal reduction of the emission may be indicative of the interaction of the antibiotic with the A chain of the toxin. Indeed, the B pentamer contains 10 tryptophans (2 in each B subunit); therefore, the NAB815-induced quenching of even one of the tryptophans in each of the B subunit would have resulted in a non-stoichiometric toxin/NAB815 ratio and likely in a much more significant reduction of the emission. Inversely, the Stx2a A chain contains 2 tryptophans out of the 12 within the holotoxin; therefore, the observed low reduction of the fluorescence emission is in good agreement with the interaction of NAB815 with the Stx2a A chain where the binding site for TLR4 is located.^12^^,^^13^ The small mass of NAB815 (MW 1,175.44) compared to Stx2a (MW 68,000) suggests the quenching of one single tryptophan of the A chain. To assess the specificity of NAB815 in interacting with Stx2a, a human protein not involved in the pathogenesis of HUS (lactate dehydrogenase A [LDH-A] containing 6 tryptophan residues) was incubated with NAB815 under the same conditions. The drug binds to the tetrameric form of LDH-A with very low affinity (K_d_ = 1 × 10^−5^ M) forming a 1:1 complex with the monomer (Figures S2A–S2C). The calculated dissociation constant is 220-fold higher than that measured with the NAB815/Stx2a complex demonstrating a clear difference in the affinity of NAB815 for the two proteins.

### NAB815 inhibits the formation of white blood cell/platelet aggregates by Stx2a

The effect of NAB815 on the formation of neutrophil/platelet or monocyte/platelet aggregates in human blood samples treated for 4 h at 37°C with Stx2a (1 nM) is shown in a representative experiment with blood samples from a single donor (Figures S3A and S3B) or by comparing different donors (Figures S3C and S3D). The inhibiting effect of NAB815 is evident (inhibition by more than 70%, Figures S3A–S3D) even at very low concentrations (0.01 μg/mL; ∼7-fold molar excess relative to Stx2a). According to previously reported results, polymyxin B is ineffective at these concentrations (Figures S3A–S3D). Also shown are the negative controls carried out for bacterial endotoxin (LPS, 0.01 EU) contaminating Stx2a preparations and with heat-denaturated Stx2a, which did not stimulate the formation of aggregates.

### NAB815 slightly affects the viability of human leukocytes but has no effect on erythrocytes

The effect of NAB815 on the viability of leukocytes after 4-h challenge was measured by assessing the annexin V binding to phosphatidylserine exposed on the outer leaflet of the plasma membrane of apoptotic cells and by evaluating, simultaneously, the exclusion of 7-aminoactinomycin D by live cells. The drug induced a dose-dependent drop in live cells, which is associated with an increased number of apoptotic cells, while the number of necrotic cells was negligible (Figure S4A). At the lower NAB815 concentration (0.01 μg/mL), the effect on leukocytes’ viability is modest; therefore, this concentration of drug was used in the following experiments. When isolated erythrocytes were similarly challenged with NAB815, no hemolysis occurred, unlike treatment with Triton X-100, chosen as a positive control (Figure S4B).

### NAB815 inhibits the formation of platelet-derived and leukocyte-derived pathogenic EVs containing Stx2a

To mimic what happens in STEC-infected patients when Stx2a enters the circulation and interacts with blood cells inducing the formation of pathogenic EVs, whole blood from healthy donors was incubated for 4 h at 37°C with Stx2a (2 nM) in the presence or in the absence of 0.01 μg/mL of NAB815, a concentration of the antibiotic that was effective in inhibiting the formation of leukocyte/platelet aggregates. At the end of the incubation, large EVs sedimenting between 10,400 and 20,800 g were isolated by centrifugation as previously performed in patients during HUS development.^20^^,^^22^

**Figure 3:**
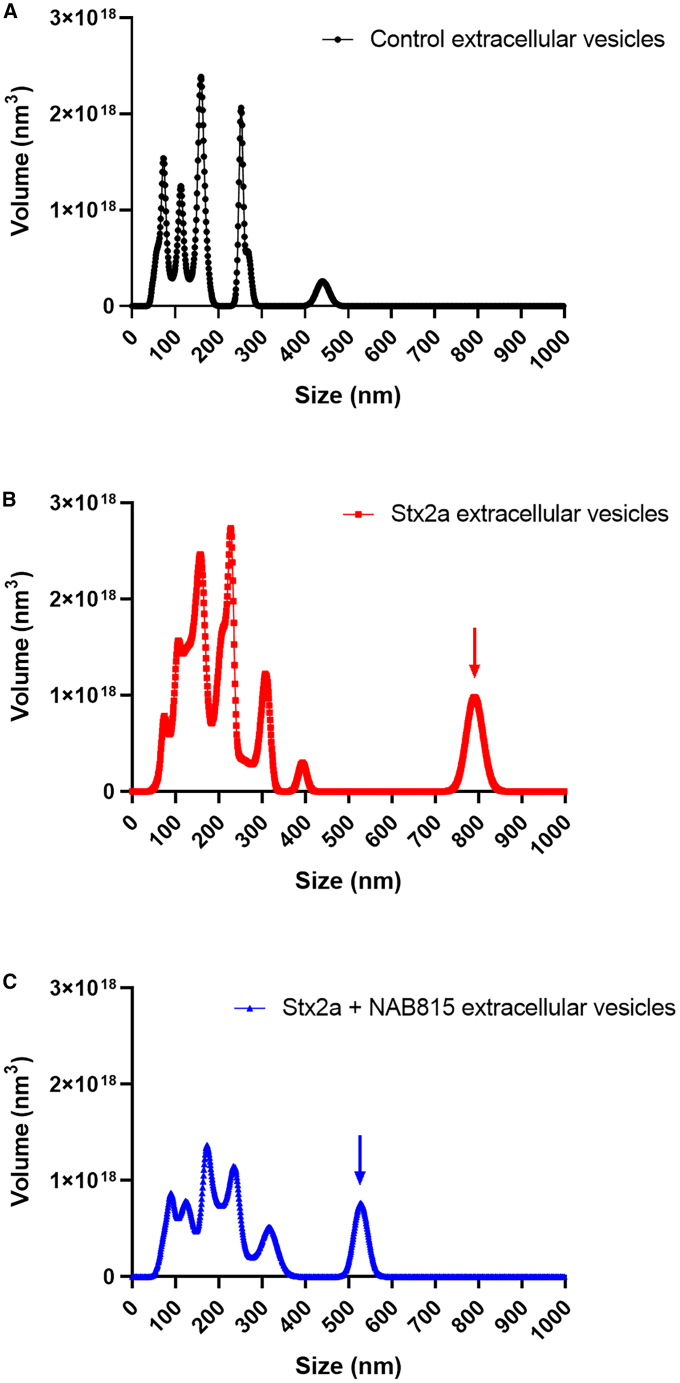
Nanoparticle tracking analysis of human EVs (A–C) Human EVs obtained after treatment of human blood from a representative donor with 2 nM Stx2a in the absence (B, red line) or in the presence (C, blue line) of 0.01 μg/mL NAB815 or vehicle (A, black line) were analyzed by nanoparticle tracking analysis. The vesicles were isolated by differential centrifugation as described in STAR Methods, 10^6^-fold diluted and applied to NanoSight. The data reported in Figure S5 (number of particles/mL) were used to calculate the total volume (nm^3^) of each population of EVs according to their diameter, assuming a spherical shape. See also Figures S5 and S6.

Nanoparticle tracking analysis of these EVs based on their Brownian motions in solution was carried out. In control samples, the presence of discrete peaks between 60 and 160 nm diameters and of a minor peak at 250 nm diameter was observed (Figure S5A, black line), as well as the occurrence of a minor 440-nm-diameter peak (Figure S5B, black line). This pattern changed in the presence of Stx2a, which induced (1) a shift toward higher values of vesicle size, (2) the appearance of a new peak at 225 nm diameter (Figure S5A, red line), and, strikingly, (3) the emergence of an additional minor component at ∼800 nm diameter (Figure S5B, red arrow). This analysis refers to the number of vesicles; if, instead, the total volume of the different vesicle populations is calculated (Figure 3), the last emerging peak at 800 nm accounts for 13% of the total mass of EVs found in Stx2a-stimulated samples (Figure 3B, red arrow). The presence of NAB815 (Figures S5 and 3, blue lines) strongly reduced the number and the total volume of EVs compared to those produced by the action of Stx2a alone, clearly inducing a size decrease of the component featuring a ∼800 nm diameter (Figures S5B and 3C, blue arrows).

To assess whether Stx2a was mainly associated with the newly emerging larger EV components and to better elucidate the effect of NAB815, we sought to perform an analytical gel filtration on selected EV samples. Aliquots of control, Stx2a, and Stx2a+NAB815 EVs were analyzed on a Sephacryl S-500 gel-filtration column (exclusion size 200 nm, 5.5 mL volume) (Figure S6). The resin allowed the exclusion in the void volume of the largest components (>200 nm diameter) found in the preparation of vesicles isolated from Stx2a-triggered blood, whereas those vesicles having diameters lower than 200 nm were separated during chromatography. In the sample containing vesicles from Stx2a-treated blood, we observed a protein peak corresponding to the void volume (Figure S6B, fraction 7, black line) and thus to the 800- and 225-nm-diameter components. Most of the toxic activity (orange circles) detected by the Vero cell translation assay was recorded in the void volume indicating that Stx2a is mainly associated, as expected, with the highest size components. Control samples devoid of Stx2a did not exert any translation inhibition (Figure S6A). In the presence of NAB815 (Figure S6C), the highest mass Stx2a-bearing EVs were greatly reduced and resulted in less toxicity for Vero cells.

**Figure 4:**
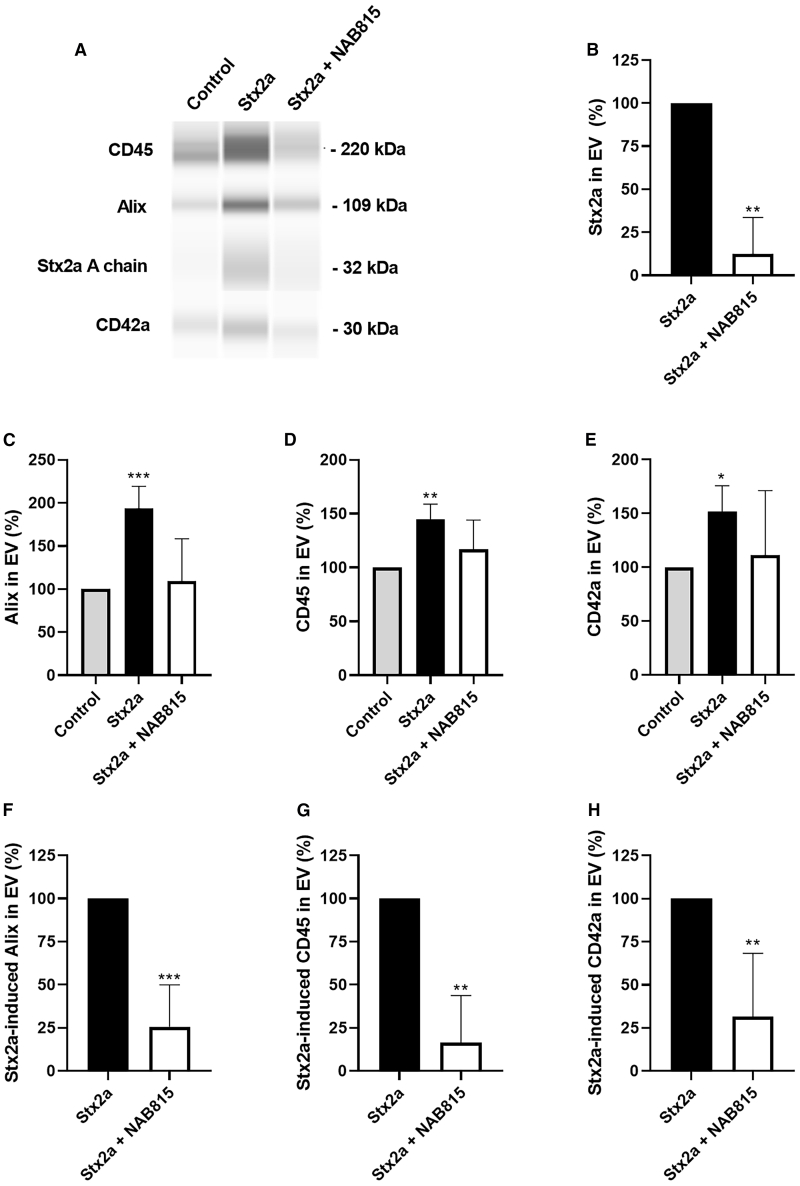
Quantitative capillary western blot analysis of the proteins derived from human EVs Human EVs were isolated after treatment of human blood with vehicle or with 2 nM Stx2a in the absence or in the presence of 0.01 μg/mL NAB815 and their proteins extracted as described in STAR Methods. (A) Representative WES of the analyzed antigens (Alix, CD45, and CD42a) and associated proteins (Stx2a) is shown. (B–E) Data obtained with different human donors (*n* = 4) represent the percentage (mean ± SD) of the quantitative determinations of the different antigens with respect to controls (C–E) or Stx2a (B). (F–H) Data obtained with different human donors (*n* = 4) are expressed as percentage (mean ± SD) of the stimulation in the presence of Stx2a that was set at 100%. Different primary antibodies were used: rabbit polyclonal anti-Alix 1:50 (Novus Biological) as an EV marker, mouse monoclonal anti-CD45 1:250 (BD Transduction Laboratories) as a leukocyte marker, rabbit polyclonal anti-CD42a 1:10 (GeneTex) as a platelet marker, and rabbit polyclonal anti-Stx2a 1:50 (Dr. Stefano Morabito, ISS Rome) to detect the toxin. ∗*p* < 0.05, ∗∗*p* < 0.01, ∗∗∗*p* < 0.001 (two-tailed unpaired t test). See also Figure S7.

Further characterization of EVs was obtained by analyzing the protein component extracted from the EV preparations by quantitative capillary western blot (WES). The results showed that in toxin-stimulated samples, the expression of the EV marker Alix doubled compared to the controls (193.4% ± 25.8%, *p* < 0.0005) (Figures 4A and 4C). Regarding the origin of the vesicles produced after toxin stimulation, a strong increase in the leukocyte antigen CD45 (144.6% ± 14.3%, *p* < 0.01) and in the platelet antigen CD42a (151.8% ± 24.0%, *p* < 0.05) was observed (Figures 4A, 4D, and 4E). NAB815 induced a strong reduction of the expression of Alix confirming the observed drop in the number of EVs (Figure 4A). When the expression due to Stx2a action was set at 100%, a residual stimulation equal to 25.7% ± 24.3% (*p* < 0.001) was observed (Figure 4F). In addition, the drug is effective in reducing the formation of platelet-derived and, especially, leukocyte-derived EVs (Figure 4A), inducing a residual stimulation of 16.4% ± 27.4% (*p* < 0.01) for CD45 and 31.6% ± 36.7% (*p* < 0.01) for CD42a (Figures 4G and 4H). NAB815 added to human blood did not significantly change the basal release of human blood cell-derived EVs (Figure S7A). Most importantly, Stx2a was found by WES in EVs derived from blood treated with the toxin, and its amount was strongly reduced when the toxic treatment was performed in the presence of NAB815 (residual percentage: 12.3% ± 21.3%, *p* < 0.01) (Figures 4A and 4B).

To sum up, the different analyses clearly show that Stx2a was mainly associated with larger EVs and that their toxic cargo was strongly reduced in the presence of NAB815.

### NAB815 impairs the recruitment of complement factors in Stx2-induced EVs

The representative WES analysis depicted in Figure S7B shows the recruitment of complement factors C3 and C9 on the EVs produced when human blood is treated with Stx2a. In the presence of NAB815, the amounts of vesicle-associated complement factors were greatly reduced and paralleled those observed in control EVs. C9 was recruited after toxin stimulation in 100% of the analyzed donors (*n* = 4), showing a mean increase with respect to control EVs equal to 158.1% ± 32.8%, *p* < 0.05. By setting at 100% the amount of vesicle-associated C9 due to Stx2a action, the residual recruitment in the presence of NAB815 was 8.1% ± 16.1% (*p* < 0.0001). Contrariwise, C3 was associated with Stx2a-induced EVs in 50% of donors, giving a 197.3% ± 44.7% (*n* = 2) mean increase compared to control vesicles. In these cases, the Stx2a-dependent binding of C3 to EVs was completely inhibited by NAB815. In conclusion, not only does the drug reduce the toxic cargo (Stx2a content) of EVs but also the recruitment of additional lytic factors involved in the pathogenesis of HUS (complement factors).

### NAB815 affords protection against toxicity induced by Stx2a-triggered EVs in cultured cells

**Figure 5:**
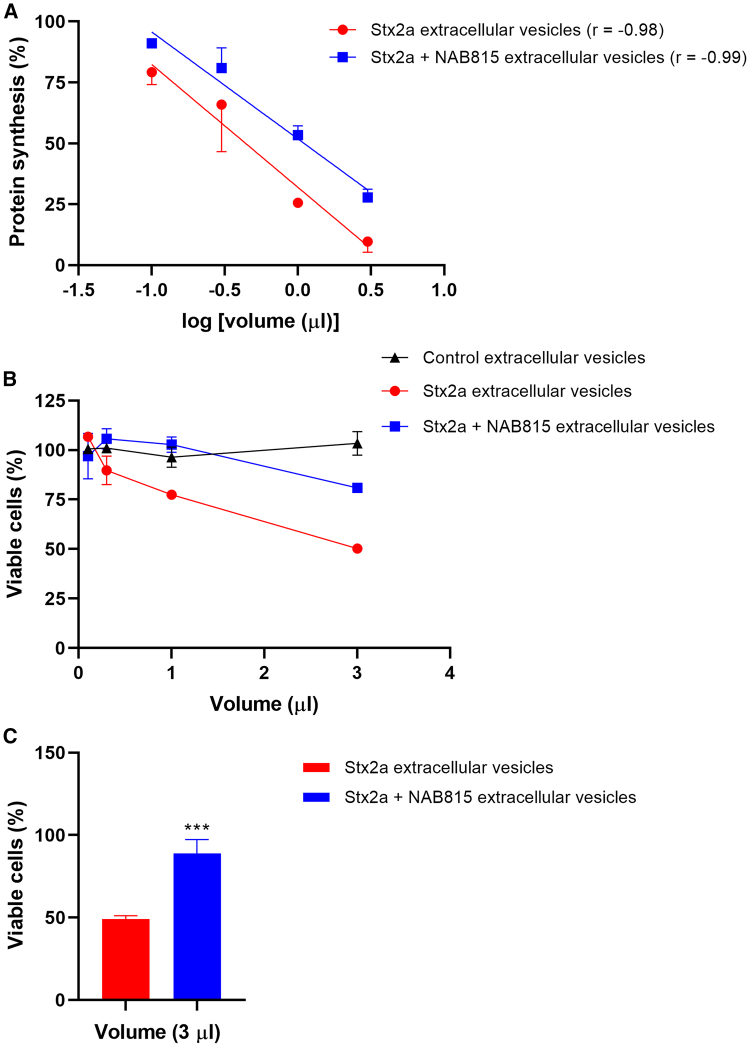
Toxicity of human EVs for Vero cells Human EVs were isolated after treatment of human blood with vehicle or with 2 nM Stx2a in the absence or in the presence of 0.01 μg/mL NAB815, and different aliquots of the preparations were added to Vero cells. The toxic effects were measured by the translation (A) and viability (B–C) assays described in STAR Methods. (A) Data are expressed as the mean percentage of protein synthesis ± SD with respect to controls carried out without vesicles obtained from human donors (*n* = 2); the reported straight lines significantly differed in the *y*-intercept (*p* < 0.05); the Pearson correlation coefficient (*r*) was used to assess the correlation between variables. (B) Data are expressed as mean percentage of viable cells ± SD with respect to controls carried out without vesicles obtained from human donors (*n* = 2). (C) Data are expressed as mean ± SD of the percentage of viable cells with respect to controls carried out without vesicles obtained from human donors (*n* = 4). ∗∗∗*p* < 0.001 (two-tailed unpaired t test).

Since Stx2a is a potent inhibitor of translation, we inferred that EV-associated Stx2a would also act as a protein synthesis inhibitor whose effect might be dampened by NAB815. Aliquots of control EVs, Stx2a-triggered EVs, and Stx2a+NAB815-triggered EVs (0.1, 0.3, 1, and 3 μL) from a representative donor were added to cultured Vero cells, and, after 20 h, the incorporation into proteins of a radioactive amino acid was measured during a 1-h incubation. Control vesicles did not affect protein synthesis, while Stx2a-triggered EVs were more toxic than those obtained in the presence of the toxin and NAB815, as indicated by the comparison of the curves obtained under the two conditions (Figure 5A). Calculation of the EV volumes that were required to obtain 50% reduction of translation (50% inhibition dose [ID_50_]) allowed determination of the number of ID_50_ in each preparation (608 in Stx2a-triggered EV preparation and 242 for Stx2a+NAB815-triggered EV preparation) giving a quantitative measure of the difference in toxicity (2.5-fold) and demonstrating the protective effect induced by the drug.

To assess whether the EV preparations by the same representative donor also induced cell lethality, the same aliquots of control EVs, Stx2a-triggered EVs, and Stx2a+NAB815-triggered EVs (0.1, 0.3, 1, and 3 μL) were used to challenge Vero cells for 72 h, and then a vitality assay was performed. Control EVs were nontoxic while Stx2a-containing EVs induced approximately 50% cell death at the highest assayed volume (3 μL), and, consistently, NAB815 provided protection (Figure 5B). Finally, a fixed volume (3 μL) of the EV preparations obtained from 4 different donors by incubating blood samples with Stx2a in the absence and in the presence of the drug was used in the vitality assay (Figure 5C). The protective effect afforded by NAB815 was significant, as the approximately 50% lethality induced by Stx2a-triggered EVs turned out to be only ∼15% in the presence of the drug (Figure 5C).

### NAB815 affords protection against free Stx2a in CD-1 mice

**Figure 6:**
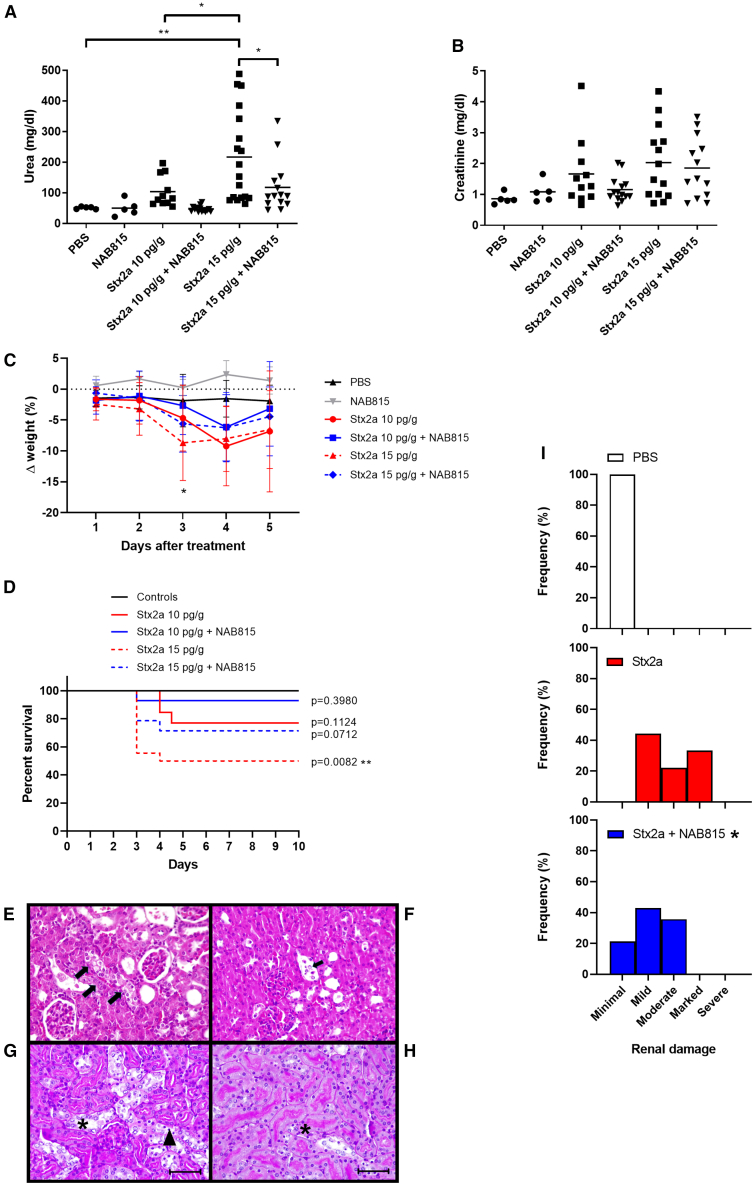
Effect of NAB815 on the intoxication of CD-1 mice with free Stx2a (A) Serum urea concentrations. (B) Serum creatinine concentrations. (C) Percentage of weight. (D) Percentage of survival change. (A–D) CD-1 mice treated with 10 and 15 pg/g of free Stx2a in the absence (*n* = 13 and *n* = 18, respectively) or in the presence (*n* = 14 and *n* = 14, respectively) of 0.01 μg/mL NAB815 and compared with control mice (*n* = 5) or NAB815-treated mice (*n* = 5). Bars in (A) and (B) represent mean values; ∗*p* < 0.05, ∗∗*p* < 0.001 (one-way ANOVA with Tukey’s multiple comparison test). In (C), data are represented as mean ± SD; a significant difference with respect to the vehicle was found in the group treated with 15 pg/g Stx2a by one-way ANOVA with Dunnett’s multiple comparison test, ∗*p* < 0.05. In (D), comparison of survival curves was performed by log rank (Mantel-Cox) test; the *p* values vs*.* controls (PBS + NAB815-treated mice, *n* = 10) have been calculated. (E) Histopathological analysis: mouse, kidney, 10 pg/g Stx2a: the arrows indicate a tubule in which the epithelial cells have increased in volume and with clear cytoplasm (hydropic degeneration). Hematoxylin-eosin, 40×. (F) Histopathological analysis: mouse, kidney, 10 pg/g Stx2a: the arrow indicates desquamated cells within the tubule. Hematoxylin-eosin, 40×. (G) Histopathological analysis: mouse, kidney, 15 pg/g Stx2a: in the distal tubules, hydropic degeneration (asterisk) is evident; the arrowhead indicates some pycnotic nuclei. PAS (scale bars, 50 μm). (H) Histopathological analysis: mouse, kidney, 15 pg/g Stx2a: pyknotic nuclei (asterisk). PAS (scale bars, 50 μm). (I) Histopathologic scoring of renal damage was performed on mice treated with PBS (*n* = 5) or intoxicated with 15 pg/g of Stx2a in the absence (*n* = 18) and in the presence (*n* = 14) of NAB815 by evaluating acute tubular injuries (ATIs) on PAS-stained kidney sections; minimal: <10% of parenchyma; mild: 11%–25%; moderate: 26%–50%; marked: 51%–75%; severe: >75%; ∗*p* < 0.05 Stx2a vs. Stx2a+NAB815 (by two-tailed unpaired Mann-Whitney t test).

The protective effect of NAB815 was also assessed in CD-1 mice intoxicated with Stx2a (10 and 15 pg/g). After 3 days, renal function was assessed by measuring serum urea and creatinine levels, and animals were further observed for 7 days. In this experimental setting, the release of endogenous EVs after toxin challenge of murine circulating cells has been previously demonstrated.^22^ When the serum urea values of the different groups were compared (Figure 6A), the protective effect of NAB815 was shown to be significant at the highest Stx2a dose. Creatinine levels non-significantly paralleled the results obtained with serum urea determinations (Figure 6B). A significant drop in body weight was observed on day 3 (before the lethal effects) in animals treated with Stx2a, while those treated with Stx2a in the presence of NAB815 did not show significant changes compared to controls (Figure 6C). These observations were corroborated by the reduced trend in lethality observed in the groups treated with Stx2a in the presence of the drug (Figure 6D) although comparison between curves did not reach statistical significance. The 10 pg/g Stx2a dose roughly corresponded to the LD_30_, while the same dose administered in the presence of the drug induced the death of a single animal showing congested bowel (on gross examination). This presentation is atypical since neither Gb3Cer intestinal expression^42^ nor accumulation of labeled Stx in the gut^44^ nor evidence of Stx-induced intestinal damage^45^ was previously observed in mice. When animals were treated with 15 pg/g Stx2a, the LD_50_ was obtained, and the protective effect induced by NAB815 was confirmed since the lethal effects observed in the group with toxin were significantly different with respect to controls only in the absence of NAB815 (Figure 6D). Kidney histopathology analysis showed the occurrence of tubular damage in animals intoxicated with Stx2a, as found in other studies with CD1 mice.^45^ Hydropic degeneration in epithelial cells (Figure 6E), detachment of cells in tubules (Figure 6F), atrophy and necrosis of epithelial cells, and ectasia of tubular lumens were present in mice intoxicated with the lower dose of toxin. PAS staining was used to distinguish proximal tubules (brush well evident) from distal tubules, in mice intoxicated with 15 pg/g Stx2a. The distal tubules showed hydropic degeneration (Figure 6G), while coagulative necrosis was present mainly in the proximal tubules (Figure 6H) in which basal membranes or cellular details are not always visible, and many nuclei are pycnotic or dissolved (karyolysis). Blinded histopathologic scoring performed by assigning different categories depending on the severity of the observed kidney damage (Figure 6I) showed significant differences between mice intoxicated with the LD_50_ of the toxin and those treated with Stx2a in the presence of the drug.

### NAB815 affords protection against EV-associated Stx2a in CD-1 mice

**Figure 7:**
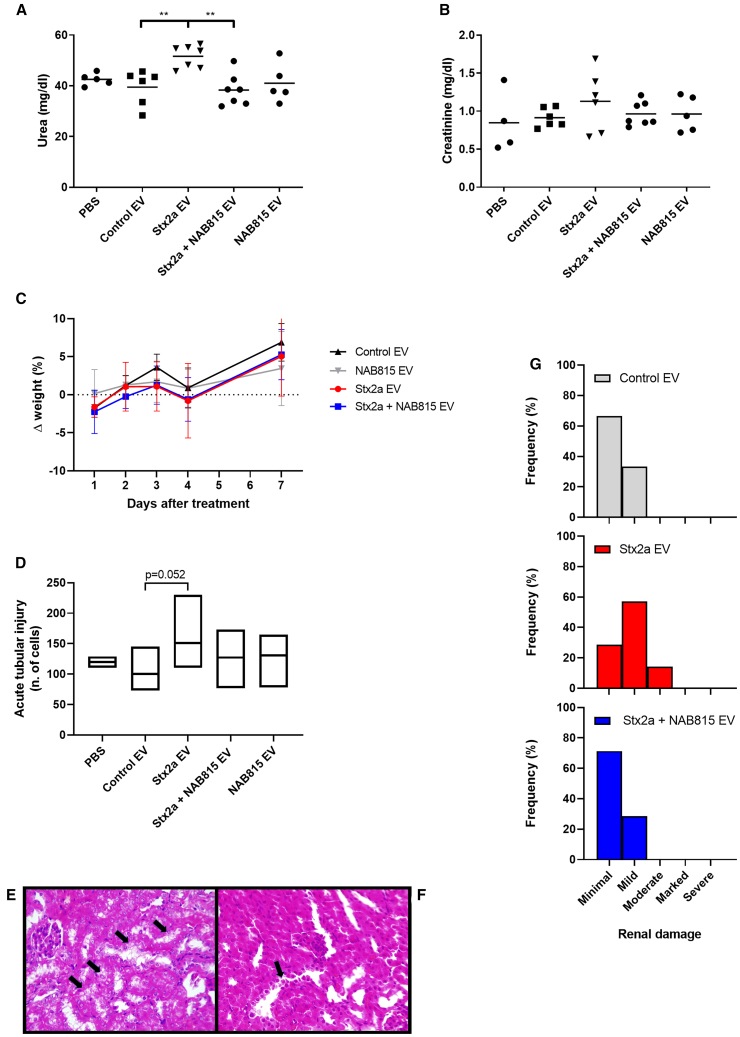
Effect of NAB815 on the intoxication of CD-1 mice with Stx2a-containing EVs (A) Serum urea concentrations. (B) Serum creatinine concentrations. (C) Percentage of weight change. (D) Acute tubular injuries. (A-D) CD-1 mice treated with PBS (*n* = 5) or with human EVs isolated as described in STAR Methods after treatment of human blood with vehicle (*n* = 6) or with 2 nM Stx2a in the absence (*n* = 7) or in the presence (*n* = 7) of 0.01 μg/mL NAB815. Bars in (A) and (B) represent mean values; ∗∗*p* < 0.01 (one-way ANOVA with Tukey’s multiple comparison test). In (C), data are represented as mean ± SD. In (D) Acute tubular injuries (ATIs) were evaluated as described in STAR Methods; the exact *p* value of the comparison Stx2a EVs vs*.* control EVs is indicated (one-way ANOVA with Dunnett’s multiple comparison). (E) Histopathological analysis: mouse, kidney: the arrows indicate tubules in which the epithelial cells have increased in volume and with clear cytoplasm (hydropic degeneration). Hematoxylin-eosin, 40×. (F) Histopathological analysis: mouse, kidney: the arrow indicates desquamated cells within the tubule. Hematoxylin-eosin, 40×. Both mice were treated with Stx2a-containing EV and analyzed after 10 days. (G) Histopathologic scoring of renal damage was performed on mice treated with control EVs (*n* = 6) or with Stx2a EVs (*n* = 7) or Stx2a+NAB815 EVs (*n* = 7) by evaluating ATI on PAS-stained kidney sections; minimal: <10% of parenchyma; mild: 11%–25%; moderate: 26%–50%; marked: 51%–75%; severe: >75%.

Since the expression pattern of Gb3Cer and TLR4 receptors of murine and human circulating cells differs considerably and considering the lack of the Stx2a-modulating factor HuSAP in mice, an animal model was set up based on the intravenous injection to CD-1 mice of the human EVs (10,400–20,800 g) described earlier. CD-1 mice were treated with 5 ID_50_ (Vero cell viability assay) per gram of pooled EVs isolated from Stx2a-treated blood obtained from 8 donors. Equal volume of vesicles deriving from control blood, NAB815-treated blood, or Stx2a+NAB815-challenged blood was administered for comparison. Although no lethal effects were detected, mice challenged with EVs from human blood treated with Stx2a exhibited increased serum creatinine and a significant rise in serum urea (Figures 7A and 7B). No changes in serum creatinine and urea were found in the groups treated with control or NAB815 vesicles. The drug was highly effective in preventing renal impairment when the administered EVs were isolated from blood treated with Stx2a in the presence of NAB815, as demonstrated by the drop of serum urea levels to those observed in the control group (Figure 7A). The same trend was observed with serum creatinine values, although differences were not significant (Figure 7B). No appreciable weight differences were observed by comparing the different groups (Figure 7C) according to the mild pathologic serum urea levels observed. At the end of the experiment (day 10), kidney histopathology showed tubular lesions similar to those obtained when animals were treated with free Stx2a (Figures 7E and 7F). The histopathologic scores obtained in mice treated with Stx2a EVs or Stx2a+NAB815 EVs (Figure 7G) showed a non-significant protective trend. Compared to controls, an increasing number of cells with acute tubular injuries (ATIs) was found in animals intoxicated with vesicle-bearing Stx2a alone but not in the other groups (Figure 7D), thus confirming that the treatment with NAB815 is protective.

In conclusion, both mouse models clearly indicated that a drug capable of perturbing the interaction of Stx2a with TLR4 is effective in preventing renal damage despite the lack of effects on the interaction between the toxin and Gb3Cer.

## Discussion

The concentration of Stx2 in the blood of STEC-infected patients before the onset of HUS has been measured by different methods, as previously reviewed.^3^ The blood concentration of Stx2 bound to neutrophils varies between 0.2 and 2 nM, while that of serum Stx2 ranges between 30 and 90 pM, a fraction of which was found to be associated to EVs in patients developing HUS.^3^^,^^20^ We have experimentally challenged human blood with 2 nM Stx2a, the maximal neutrophil-associated Stx2 blood concentration, to allow toxin binding to circulating cells and subsequent release of pathogenic EVs.

In our experimental setting, Stx2a added to human blood increased the release of blood cell-derived EVs (10,400–20,800 g), similar to those found in patients with HUS or in STEC-infected children the day before the onset of the syndrome. Among the different EVs found in our preparations, the larger components (>200 nm diameter) separated by analytical gel filtration chromatography were found to be highly toxic to eukaryotic cells sensitive to Stx2a. It should be noted that Stx2a (68 kDa) or the largest complex HuSAP-Stx2a (930 kDa), if present, should not have been eluted in these fractions since the latter complex, if globular, would have a ∼13 nm diameter (according to the equation r = 0.066 × mass^1/3^),^46^ well below the exclusion limit of the resin. These observations suggest that Stx2a is associated with the larger EVs, hence mimicking what happens in the blood of STEC-infected patients before the onset of HUS.

The characterization of EVs showed that Stx2a added to human blood stimulates the release of new (Alix+) vesicles of platelet (CD42a^+^) and leukocyte (CD45^+^) origin. These results agree with data obtained by flow cytometry on EVs isolated from the blood of STEC-infected children with HUS. The majority of these vesicles were platelet derived (50%), followed by leukocyte-derived vesicles (34%), 18% and 16% of which were from monocytes (CD38^+^) and neutrophils (CD66^+^), respectively.^22^

In this article, we have identified the antibiotic NAB815 as an inhibitor of the binding of Stx2a to TLR4 expressed by human circulating cells. This compound is a derivative of polymyxin B, a well-known inhibitor of the interaction between LPS and TLR4 expressed by monocytes and macrophages,^29^^,^^30^^,^^31^ and also of the binding of Stx2a to the same receptor.^32^ Herein, we have shown that, compared to polymyxin B, NAB815 inhibits the binding of Stx2a to TLR4 at tens of times lower concentrations, as low as 0.01–0.1 μg/mL. These NAB815 concentrations are non-toxic to human renal tubular cells (IC_50_ 334 μg/mL)^37^ and are sub-bactericidal. In a study comprising 111 clinical *E. coli* isolates, the MIC_90_ of NAB815 was 4 μg/mL,^37^ the MIC_50_ was 2 μg/mL, and the range was 1–64 μg/mL (unpublished results).

NAB815 binds to Stx2a (K_d_ = 5 × 10^−8^ M, 1:1 stoichiometry), preventing its interaction with TLR4-expressing cells and reducing (1) the formation of neutrophil-platelet or monocyte-platelet aggregates and (2) the production of toxin-containing EVs, both platelet and leukocyte derived, in human blood challenged with Stx2a. By nanoparticle tracking analysis, NAB815 was shown to considerably reduce the formation of a specific population of large EVs (>200 nm diameter) produced by the action of Stx2a. In addition, WES clearly showed a strong reduction of Alix, CD42a, and especially CD45 expression in the presence of the drug. The almost total inhibition of leukocyte-derived vesicles (CD45^+^) can be explained by the fact that one of the circulating cells that interact with Stx2a (i.e., neutrophils) does not express the Gb3Cer receptor and can only bind the toxin through TLR4. In the case of platelets, the inhibition of vesicle formation is lower, albeit considerable, although these cells also express Gb3Cer, and this highlights the relevance of TLR4 in the formation of pathogenic EVs involved in HUS pathogenesis.

Remarkably, NAB815 strongly reduced the amount of Stx2a associated with Stx2a-triggered EVs, as demonstrated by WES analysis. These findings have been confirmed in cellular models since treatment of sensitive cells (Vero cells) with vesicles derived from Stx2a-treated blood induced a significant inhibition of protein synthesis leading to lethal effects that were greatly reduced in the presence of NAB815. The beneficial effect on cell viability induced by NAB815 at 72 h was observed even if protein synthesis was considerably inhibited at 20 h, although less than in the absence of the drug. This was likely due to the more rapid recovery of cells in which translation was less impaired.

To prove the efficacy of NAB815 *in vivo*, we have exploited two different animal models based on female CD-1 mice. The first model has been used in several laboratories^44^^,^^45^^,^^47^^,^^48^ and is based on intravascular treatment of mice with Stx2a. Noticeably, NAB815 strongly impaired the action of non-vesicle-bound Stx2a when the toxin was administered to animals at 10–15 pg/g. The drug reduced the extent of acute renal failure, assessed by serum urea and creatinine concentrations, and dampened the toxin-induced lethal effects.

In the second model, we sought to reproduce what occurs in the blood of STEC-infected patients when Stx2a-containing EVs are released in the bloodstream and challenge the kidney. Mice were injected directly with the human-derived EVs described earlier, which resulted in toxicity when they were isolated from human blood treated with Stx2a. The presence of NAB815 during human blood treatment with Stx2a clearly induced protection, as indicated by serum urea and creatinine levels changing from pathologic (treatment with Stx2a) to normal (treatment with Stx2a+NAB815) values.

The pathologic serum urea median concentrations obtained in mice treated with free Stx2a were higher than those found in animals treated with Stx2a-triggered EVs. Since animal treatment was performed several weeks after the preparation of pathogenic EVs, we argue that storing the EVs at −80°C in PBS may have reduced their toxicity to mice. Nevertheless, in both animal models, we found that lethality or intoxication correlated better with serum urea values rather than serum creatinine concentrations. It should be considered that pathologic serum creatinine levels are mainly influenced by kidney impairment, if diet intake and muscle activity are similar among animals, while pathologic serum urea levels, besides being influenced by glomerular function, are also affected by tubulointerstitial dysfunction. Accordingly, in mice, the renal Gb3Cer receptor targeted by Stx2a (and by Stx2a-containing EVs) is confined to tubular cells, while in humans, it is expressed by both glomerular endothelial cells and renal tubular cells, besides podocytes and mesangial cells.^42^ Consistently, renal histopathology revealed only tubular injuries in Stx2a-treated or Stx2a-containing EV-treated animals.

As regard the experiments with free Stx2a, it was completely unexpected that a drug tailored to prevent the interaction of Stx2a with TLR4 on circulating cells while allowing the binding to the specific Gb3Cer receptor on target cells would be able to reduce toxicity in mice. It should be noted that TLR4 is considered a co-receptor, which facilitates the intoxication of target Gb3Cer-expressing cells by Stx. Indeed, human umbilical endothelial cells or cancer cells showed reduced Stx binding upon small interfering RNA-mediated TLR4 depletion.^49^ A possible explanation of the protective effect induced by NAB815 in mice is the impairment of free Stx2a-TLR4 interactions during the intoxication of renal tubular epithelial cells that express TLR4 besides Gb3Cer.^50^ However, NAB815 was virtually absent when EVs were inoculated into mice due to the washing procedures performed during their preparation. Moreover, when free Stx2a was intravenously injected into mice in association with NAB815 at 0.01 μg/mL (100-μL volume), the drug was immediately diluted in the blood of mice (∼1.3 mL in a 20-g mouse^51^), hence reducing ∼10-fold the concentration of the drug. It seems unlikely that this very low concentration of the drug would be able to prevent the role of TLR4 as a co-receptor. In this light, the most likely explanation of the phenomenon is that, even in the mouse, the most dreadful form of the toxin is that associated with EVs produced by murine blood cells rather than free Stx2a. This is even more surprising given that there are no naturally occurring serum inhibitors of free Stx2a in mice as in the case of HuSAP, in humans. Indeed, in mice, contrariwise to humans, free Stx2a has two options to target the kidney, i.e., (1) by inducing the release of blood cell-derived EVs and (2) by interacting directly with renal target cells. Only the first way is preventable by NAB815; hence, in mice, a portion of free Stx2a is able to directly hit the target. Despite this, NAB815 turned out to be effective in protecting Stx2a-intoxicated mice. In humans, the second way is naturally prevented by HuSAP. Therefore, we argue that in humans, in whom Stx2a is forced to circulate in vesicle-bound form given the strong inhibition of free toxin action by HuSAP, the drug NAB815 might be even more effective than in mice. In addition, an effect of NAB815 in reducing the binding of Stx2a to TLR4 acting as a co-receptor during the intoxication of renal target cells might be envisaged when the drug will be therapeutically administered thrice a day to reach the working plasmatic concentrations (0.01 μg/mL). Further evidence supporting these conclusions might be obtained by using animal models closer to humans, such as baboons, and/or by planning clinical studies on NAB815’s efficacy in preventing HUS in STEC-infected patients. In some countries, specific surveillance systems have been developed allowing early diagnosis of STEC infections in children with bloody diarrhea. Early diagnosis and prompt therapeutic intervention are necessary prerequisites for the development of appropriate clinical studies.

Today, there are no specific effective therapies for STEC infections, and management of patients relies on supporting treatments: fluid infusion, kidney replacement therapy, and packed red cells transfusions.^4^ Plasma exchange and the use of specific inhibitors of the complement system are not currently recommended.^4^ It should be noted that most studies have shown that treatment of patients with antibiotics should be avoided as they potentially increase the risk of developing HUS by activating bacterial SOS response, which leads to enhanced Stx expression and release after bacterial lysis.^4^^,^^26^^,^^27^ Antibiotics used against *E. coli* and belonging to different chemical lineages are usually characterized by divergent minimum inhibitory concentrations (MICs) and by dissimilar SOS-inducing concentrations (SICs). Accordingly, gentamicin and mitomycin C represent compounds featuring poor and strong SOS-inducing actions, and notably, ciprofloxacin is an antibiotic frequently used as a model antibiotic inducing the SOS response. As to the MIC/SIC ratio, the estimates were equal to 1.8, 1.4, and 8.^52^^,^^53^^,^^54^ Therefore, it seems reasonable to assume that using an antibiotic at a concentration two orders of magnitude lower than its MIC should not induce any SOS response in *E. coli*. In addition, it should be considered that polymyxin B (the parental compound of NAB815) was tested on quite a number of *E. coli* human isolates, and this analysis revealed consistent MIC values, i.e., 1 μg/mL.^55^ Quite recently, the same antibiotic was found to elicit the SOS response when used at concentrations higher than 1 μg/mL.^56^ Consistently, polymyxin B was shown to induce the release of considerable amounts of Stx by Shigella only at concentrations >1 μg/mL.^57^

A recent approach based on early volume expansion has shown to reduce progression to HUS and/or to mitigate the severity of the complication in patients with early diagnosis of STEC infection.^4^^,^^24^ However, a specific drug aimed at preventing the onset of HUS after early diagnosis of STEC infection and within the therapeutic windows is crucial. The administration of NAB815 at sub-bactericidal concentrations in patients with STEC infection could be an innovative treatment for the prevention of HUS, as Stx2a associated with EVs via TLR4 was detected in patients’ blood the day before HUS development.^20^ These promising results lay the foundation for targeted animal and clinical studies.

### Limitations of the study

Limitations of this study are related to the animal model used to demonstrate the protective effect of NAB815 on renal damage *in vivo*. The mouse model does not fully recapitulate the natural course of human STEC infections, and Stx target mainly tubular cells in mice rather than glomerular endothelial cells as in humans. The more complex and challenging baboon model should be preferred for further studies. An additional limitation is that, in our study, female mice were used, while human blood samples were from male volunteers. However, it is worth noting that, in humans, both sexes are equally affected by HUS.

## Resource availability

### Lead contact

Further information and requests for resources and reagents should be directed to and will be fulfilled by the lead contact, Maurizio Brigotti (maurizio.brigotti@unibo.it).

### Materials availability

This study did not generate new unique reagents.

### Data and code availability

• All data reported in this article will be shared by the lead contact.
• This article does not report original codes.
• Additional information needed to reanalyze the data presented in this article can be obtained from the lead contact upon request.

## Acknowledgments

This work was funded by grants from Progetto ALICE ONLUS grant 2022 (to M. Brigotti) and the University of Bologna (RFO and Proof-of-Concept funds POC2021 to M. Brigotti).

## Author contributions

E.V., L.C., D.C., E.G., B.M., E.P., M.P., G.R., B.B., F.R., P.L.T., F.M, P.P., G.S., and A.H. performed the *in vitro* experiments. F.P. and C.B. performed the *in vivo* experiments. E.V., L.C., D.C., E.G., B.M., E.P., M.P., G.R., F.P., C.B., B.B., F.R., P.L.T., F.M., I.M., P.P., G.S., M. Bonafè, A.H., A.Z., S.M., G.A., T.V., M.V., and M. Brigotti analyzed the data. M. Brigotti designed the study and wrote the manuscript. All authors read the manuscript and approved the final version.

## Declaration of interests

M. Brigotti, D.C., E.P., and E.G. are inventors on a patent related to the topic of the paper: n.102019000025414 (2019) “Compound for the treatment of hemolytic-uremic syndrome” Alma Mater Studiorum – University of Bologna; WO2021130700 (A1) (2021). T.V. is the co-owner and CEO of Northern Antibiotics Ltd., the company that developed NAB815.

## STAR★Methods

### Key resources table

**Table:** 

REAGENT or RESOURCE	SOURCE	IDENTIFIER
**Antibodies**		

Anti-CD41-PE	Beckman Coulter	Cat#A07781; clone P2
Anti-CD14-FITC	Beckman Coulter	Cat#B36297
Anti-CD16-PC5	Beckman Coulter	Cat#A07767; clone 3G8
Rabbit polyclonal anti-Alix 1:50	Novus Biologicals, Bio-Techne	Cat#NBP1-49701; RRID: AB_10011818; lot: D10 6916-8
Mouse monoclonal anti-CD45 1:250	BD Transduction Laboratories	Cat#610266; RRID: AB_397661; clone 69/CD45
Rabbit polyclonal anti-CD42a 1:10	GeneTex	Cat#GTX32502; lot:822202558
Rabbit polyclonal anti-Stx2a 1:50	Dr Stefano Morabito, ISS Rome	N/A
Mouse monoclonal anti-C3 1:10	Hycult Biotech	Cat#HM2394; clone C3-42.3
Mouse monoclonal anti-C9 1:10	Hycult Biotech	Cat#HM2264; RRID: AB_10502672; clone WU 13-15
Anti-Mouse Secondary Antibody (∗kit component)	ProteinSimple, Bio-Techne	lot: 105741
Anti-Rabbit Secondary Antibody (∗kit component)	ProteinSimple, Bio-Techne	lot: 105824

**Bacterial and virus strains**		

C600-933W strain	Department of Microbiology and Immunology, Uniformed Services University of the Health Sciences, Bethesda, MD, USA	N/A

**Biological samples**		

Human blood	Emilia-Romagna Regional Blood Centre, Maggiore Hospital, AUSL Bologna, Italy	N/A

**Chemicals, peptides, and recombinant proteins**		

Bio-Rad protein assay	Bio-Rad	Cat#500-0006
Folin-Ciocalteu’s phenol reagent	Merck	Cat#F9252
Penicillin-Streptomycin	Cambrex	Cat#DE17-602E
L-glutamine	Sigma-Aldrich	Cat#G7513
FBS	Lonza	Cat #DE14-701E
Ficoll-Paque	Merck	Cat#GE17-1440-02
Leucine, L-[4,5-3H] radionuclide	PerkinElmer	Cat#NET1166001MC
Trichloroacetic acid	Carlo Erba	Cat#411527
Fibrin polymerization inhibitor Gly-Pro-Arg-Pro (GPRP)	Merck	Cat#G1895-5MG
cOmplete protease inhibitor cocktail	Sigma-Aldrich	Cat#11836170001
RPMI 1640 medium	Lonza	Cat#BE12-702S
DMEM High Glucose (4,5 g/L), w/o L-Glutamine, with Sodium Pyruvate	Capricorn Scientific	Cat#DMEM-HPXA
polymyxin B derivative NAB741	Northern Antibiotics	N/A
polymyxin B derivative NAB7061	Northern Antibiotics	N/A
polymyxin B derivative NAB815	Northern Antibiotics	N/A
Cyanogen bromide-activated Sepharose 4B	Pharmacia	N/A
Glycoprotein B1011 (Galα1-4Galβ-O-spacer-BSA)	Glycorex AB	N/A
EZ Standard Pack 1	ProteinSimple, Bio-Techne	Cat#PS-ST01EZ-8
Anti-Mouse Detection Module ∗	ProteinSimple, Bio-Techne	Cat#DM-002; lot: 35507
Anti-Rabbit Detection Module ∗	ProteinSimple, Bio-Techne	Cat#DM-001; lot: 356039
L-Lactate Dehydrogenase (LDHA)	Sigma-Aldrich	Cat#SAE0049

**Critical commercial assays**		

ToxinSensor™ Gel Clot Endotoxin Assay Kit	GenScript	Cat#L00351
EasySep Human Neutrophil Enrichment Kit	Stemcell Technologies	Cat#18001
Cell titer-Glo® luminescent assay	Promega	Cat#G7570
Annexin V-FITC kit	Miltenyi Biotec	Cat#130-092-052
7-AAD BD- Pharmingen	BD-Pharmingen	Cat#559925
Urea assay kit	Abcam	Cat#ab83362
Creatinine assay kit	Abcam	Cat#ab65340

**Experimental models: Cell lines**		

Raji cells	Prof. Andrea Bolognesi, DIMEC, UNIBO. Bologna	N/A
Vero cells	Dr Stefano Morabito, ISS Rome	N/A

**Experimental models: Organisms/strains**		

CD-1 female mice: CD-1® IGS Mouse [Crl:CD1(ICR)], Outbred	Charles River Laboratories	Strain code: 022

**Software and algorithms**		

ReactLab™ Equilibria	Jplus Consulting Pty Ltd	N/A https://www.jplusconsulting.com/Products/
NTA 3.4 analytical software	Malvern Panalytical	N/A
GraphPad Prism 8 software	GraphPad Software	RRID:SCR_002798https://www.graphpad.com/features

**Other**		

NanoSight RS300 system	Malvern Panalytical	N/A
Wes	ProteinSimple, Bio-Techne	Cat#004-600
Spark	Tecan	N/A
Gel-filtration column Sephacryl S-500 HR	Merck	Cat#GE17-0613-01
ActiClean Etox column	Sterogene Bioseparations	Cat#2705
950 spectrophotometer	PerkinElmer	N/A
Lyric cytometer	Becton Dickinson	Cat#BDFACSLyric

### Experimental model and study participant details

#### Animals

The animal studies were approved by the Institutional Animal Care and Use Committee of the University of Bologna (Comitato per il benessere degli animali, CoBA) and by the Italian Ministry of Health with Ethics Approval Code n. 583/2022-PR on 3 October 2022. Animal experiments were conducted according to European directive 2010/63/UE and Italian laws 26/2014.

Female CD-1 strain mice (*Mus musculus*), 6–7 weeks old (21–26 g) were purchased from Charles River Laboratories and housed in the animal facilities of the Department of Veterinary Medical Sciences (University of Bologna, Italy). The animals were kept in individually ventilated cages measuring 1500 cm² each (1500U Blu Line Cages, Tecniplast S.p.A., Buguggiate, VA, Italy), with 50 air changes per hour via HEPA-filtered air and had access to feed and water ad libitum. Five-to-ten conspecifics were housed per cage to promote species-specific behavior. Enrichment materials such as cardboard tunnels, sizzle nests, and red mouse houses were provided to encourage natural behaviors. The housing facility is equipped with temperature (20°C–24°C) and relative humidity (45–65%) control, features air exchange (15–20 changes per hour) and follows a 12 h day-night cycle.

In the first model, mice received Stx2a (10 or 15 pg/g) diluted in PBS-BSA 1% in the presence or in the absence of 0.01 μg/mL of NAB815 by injecting 100 μL of the solutions into the tail vein. An equal volume of PBS-BSA 1% or of a solution containing NAB815 alone in the vehicle was administered to animals for comparison.

In the second experimental setting, mice received an intravenous injection of human EVs prepared as described below by treating human blood with 2 nM Stx2a in the presence or in the absence of 0.01 μg/mL of NAB815, or with NAB815 alone or PBS as a vehicle. The calculated dose of Stx2a administered to mice through the vesicle preparation obtained solely from blood treated with Stx2a corresponded to 5 ID_50_ (as determined by the Vero cell viability assay) per gram, which equals 15 pg/g, and was administered by injecting 300 μL of the suspension into the tail vein. Equal volumes of EVs derived from the other experimental conditions (vehicle, drug alone, toxin+drug) were administered to animals.

All solutions (first model) and suspensions (second model) were kept on ice for 1 h and brought to room temperature a few minutes before animal treatment. At 72 h post-injection, blood samples were collected from mice and serum samples were prepared for the determination of urea and creatinine concentrations (Urea assay kit, ab83362 Creatinine assay kit ab65340, Abcam) following the manufacturer’s instructions. Serum was obtained from blood collected in 1.5 mL Eppendorf tubes without anticoagulant via retro-mandibular bleeding. Samples were allowed to clot at room temperature for 30 minutes and subsequently centrifuged at 4000g for 10 minutes at the same temperature to separate the serum from the clot. Following centrifugation, the serum was carefully transferred into new tubes and stored at −20°C. Mice were weighed daily and monitored for their general condition, morbidity, and mortality for a period of 10 days after intoxication. After treatment with free Stx2a, mice lose weight (Figure 6C), likely because of dehydration, and this could induce blood volume contraction affecting the determination of urea level at day 3. Calculation of the total body water contraction according to a mean weight loss of 4.7% or 8.7% (means) for mice treated with 10 pg/g or 15 pg/g Stx2a (Figure 6C) gave contractions of plasma volume of ∼7% or ∼12%, respectively (assuming that total body fluids are ∼70% of the body weight of a 20 gr-mouse and that plasma volume is 1 mL). Since the means of serum urea level found in the same animal groups are ∼2- or 4-fold higher than those measured in control mice (Figure 6A), it seems unlikely that volume contraction could be responsible of the increased urea levels.

#### Human participants

Blood samples (400 mL) anticoagulated with CPD (citrate-phosphate-dextrose solution) were obtained from 12 healthy adult male volunteers (median age 50 years, range 19–60 years, ABO groups: 6 A1+, 4 B+, 1 A1B+, 1 O-) casually recruited in the Emilia-Romagna Regional Blood Centre (Maggiore Hospital, AUSL Bologna, Italy) and used consecutively in the study after obtaining informed consent. Blood from donors 1–4 (median age 51 years, range 24–60 years, ABO groups: 1 A1+, 1 B+, 1 A1B+, 1 O-) was used for the experiments on leukocyte/platelet aggregates, for the characterization of EVs after blood treatment with toxin and drug, and for cell translation and viability assays. Blood from donors 5–12 (median age 47 years, range 19–56 years, ABO groups: 5 A1+, 3 B+) was collected for the isolation of the EVs used in the animal experiments. The blood samples were allocated to the two experimental groups only on the basis of a temporal criterion; i.e., first the experiments on the mechanism of action of the drug were conducted (blood samples 1-4), then the experiments on animals were performed (blood samples 5–12).

#### Microbe strains

C600-933W strain, an *E. coli* K12 containing the bacteriophage carrying the Stx2a gene of the STEC EDL 933 strain, was maintained in Luria-Bertani medium and used for the production of Stx2a. The microbe strain was a generous gift of Dr. Alison O’Brien (Department of Microbiology and Immunology, Uniformed Services University of the Health Sciences, Bethesda, MD, USA).

#### Cell lines

Human Gb3Cer-expressing Raji cells (male) were kindly provided by Prof. Andrea Bolognesi (Department of Medical and Surgical Sciences, University of Bologna, Bologna, Italy) and were cultured in RPMI 1640 medium (Lonza, Walkersville, MD) containing antibiotics (60 U/mL penicillin, 60 mg/mL streptomycin; Cambrex) and supplemented with 4 mM L-glutamine (Sigma-Aldrich) and 10% FBS (Lonza). Simian (*Cercopithecus aethiops*) Gb3Cer-expressing Vero cells (female) were kindly provided by Dr Stefano Morabito (European Reference Laboratory for Escherichia coli, Istituto Superiore di Sanità, Rome, Italy) and were cultured in DMEM (Dulbecco modified essential medium, high glucose, w/o L-Glutamine, with sodium pyruvate; Capricorn Scientific) supplemented with 10% FBS, 2 mM L-glutamine and 1% penicillin-streptomycin. Cells were maintained in incubators at 37°C in a water-saturated 5% CO_2_ atmosphere in air. Cell lines, tested for mycoplasma contamination, were not authenticated.

### Method details

#### Toxins

Stx2a was produced from the above-mentioned *E. coli* strain and isolated through receptor analog affinity chromatography (1.5 × 0.6 cm-column, 1 mL-volume) with cyanogen bromide-activated Sepharose coupled to bovine serum albumin (BSA) linked to a terminal galabiose (Galα1-4Galβ-*O*-spacer—BSA, Glycorex, Lund, Sweden).^58^ Isolated Stx2a was subsequently applied to an ActiClean Etox column (Sterogene Bioseparations, Carlsbad, CA, USA) to reduce the contamination by endotoxin. The purified Stx2a was quantified by the Lowry assay and the amount of LPS (4.88 ng/mg Stx2a) assayed using ToxinSensor™ Gel Clot Endotoxin Assay Kit (GenScript, Piscataway NJ, USA).

#### Binding of Stx2a to human neutrophils

Human neutrophils (99.7% purity) isolated under sterile conditions with low endotoxin contamination were obtained from buffy coats of healthy donors (blood anticoagulated with EDTA) after centrifugation on Ficoll-Paque followed by sedimentation with dextran, hypotonic lysis of the erythrocytes and positive removal of contaminating cells using EasySep Human Neutrophil Enrichment Kit (Stemcell Technologies, Vancouver, BC, Canada).^13^ We used Eppendorf tubes pretreated with PBS (phosphate buffered saline) containing BSA 1% with low endotoxin content (≤1 Eu/mg Sigma) to avoid non-specific loss of toxin.^13^ The determination of the amount of Stx2a bound to neutrophils was carried out by adding a mouse monoclonal antitoxin IgG in the presence of human serum to avoid non-specific binding, washing with PBS and subsequent addition of an anti-murine IgG fluorescent sheep antibody (FITC). The fluorescence-based flow cytometry analysis allows for the detection of the fluorescence associated with neutrophils.^59^ The MCV (mean channel value of fluorescence) of the obtained histograms was chosen for the quantitative determination of the binding of Stx2a to neutrophils.

#### Determination of the binding of Stx2a to NAB815 by the intrinsic fluorescence of the toxin

Absorption spectra of Stx2a were recorded on a standard PerkinElmer λ950 spectrophotometer. Fluorescence spectra were obtained on an Edinburgh spectrofluorimeter with right angle detection geometry. All the spectroscopic experiments were carried out at 22°C.

The best complexation model, the binding constants as well as single spectra of the complexes were determined by means of a multivariate global analysis of multiwavelength data, analyzing the complete set of fluorescence spectra corresponding to different Stx2a/NAB815 mixtures. We used the commercial program ReactLab™ Equilibria (Jplus Consulting Pty Ltd) developed in Matlab®. Control experiments were conducted also on the tetrameric form of LDH-A that was challenged with NAB815 under the same conditions.

#### Raji cells translation

Raji cells were seeded in a 24-well plate (300,000 cells in 500 μL of medium). Protein synthesis was measured as the rate of incorporation of [^3^H]leucine into proteins during 60 min incubation in complete medium as described previously.^43^ Briefly, after incubation the content of each well was transferred to a 2 mL Eppendorf tube and centrifuged for 5 minutes at 200g at room temperature. The supernatant was removed and the cell pellet resuspended in 1 mL of 0.1 N KOH and incubated for 5 minutes at room temperature in order to cleave the bond between tRNAs and amino acids. Each sample was transferred to a 10 mL plastic tube containing 20% TCA to precipitate the proteins which were subsequently collected on glass microfiber filters (Whatman GF/C) presoaked with 10 mM cold L-leucine following filtration of the sample. The radioactivity associated with the filter was measured in a liquid scintillation β counter.

#### Vero cells translation and viability assays

Vero cells were seeded in a 24-well plate (50,000 cells in 500 μL of medium). After 24 h, the cell monolayer was treated with Stx2a or EVs and incubated for 20 h (translation assay) or 72 h (viability assay) at 37°C. Protein synthesis was measured as the rate of incorporation of [^3^H]leucine in the newly synthesized proteins in 60 minute-incubation. The medium of each well was removed and cells were washed 3 times with 1 mL PBS and 3 times with 1 mL of TCA 10%, then the precipitated proteins were solubilized in 200 μL of 0.2 N KOH and transferred in a plastic tube for radioactivity determinations in a liquid scintillation β counter. A luminescent cell viability assay (Cell titer-Glo®, Promega, Milan, Italy) was used to assess the number of live cells in culture by quantifying the amount of ATP as a product of metabolically active cells. Briefly, after incubation the medium was removed and 150 μL of fresh medium was added to each well followed by the addition of an equal volume of Cell Titer-Glo Reagent. After 2 minute-shaking to induce cell lysis, 200 μL of the mixture were transferred to a 96-well plate. After 10 minute-incubation at room temperature, a luminescent signal proportional to the amount of ATP present in the sample was recorded.

#### Detection of leukocyte/platelet aggregates

The formation of white blood cell/platelet aggregates was evaluated by direct fluorescence-based flow cytometry.^32^ After osmotic lysis of the erythrocytes, the samples were incubated with different fluorescent monoclonal antibodies: anti-CD41 labelled with phycoerythrin (PE) to detect platelets, anti-CD14 labelled with FITC to detect monocytes and anti-CD16 labelled with phycoerythrin cyanine 5 (PC5) to detect neutrophils, on the gated granular cells. Controls were carried out with the appropriate isotopic antibodies to exclude false positives. The total or granular cell populations showing a double CD14/CD41 positivity or a double CD16/CD41 positivity were identified as monocyte/platelet or neutrophil/platelet aggregates, respectively.

#### Determination of white cell survival, apoptosis and necrosis after treatment with NAB815

The viability of leukocytes was determined by using the Annexin V-FITC kit (Miltenyi Biotec) and 7-aminoactinomycin D (7-AAD, Becton Dickinson). Blood samples (1 mL) from healthy donors were treated with NAB815 for 4 h at 37°C. After osmotic lysis of the erythrocytes, 10^5^ leukocytes were centrifuged at 300g for 5 minutes and washed with PBS. Supernatant was discarded and cells were resuspended in 100 μL of PBS. Annexin V-FITC was added (10 μL) and the samples incubated for 15 minutes, then 7-AAD was added (20 μL) and incubated for the same time. Cells were washed and resuspended in 500 μL of PBS and analysed by a Lyric cytometer (Becton Dickinson) distinguishing Annexin V positive cells, double positive Annexin V/7-AAD and 7-AAD positive cells.

#### Determination of human erythrocyte hemolysis after treatment with NAB815

Blood samples (5 mL) from human donors were centrifuged at 500g for 5 min and packed erythrocytes were washed three times by adding PBS to 5 mL followed by centrifugation as above. Washed erythrocytes were resuspended in 5 mL of PBS, red cells were 50-fold diluted with PBS and incubated with NAB815 or triton X-100. After 4 h at 37°C, erythrocytes were sedimented as above and the absorbance at 540 nm of the supernatant was recorded.

#### Isolation and analysis of blood cell-derived EVs

Whole blood from healthy donors (80 mL) diluted with an equal volume of DMEM and supplemented with the fibrin polymerization inhibitor Gly-Pro-Arg-Pro (GPRP, 10 μM; Merck) was incubated 4 h at 37°C with 2 nM Stx2a or NAB815 (0.01 μg/mL) or Stx2a+NAB815 or vehicle. Then, EVs were isolated by differential centrifugation; i.e., 2600g for 35 min to remove blood cells and platelets, 10,400g for 25 min to remove cell debris and apoptotic bodies, 20,800g for 1 h to sediment larger EVs. In the case of donors 1–4, each pellet was resuspended in 1 mL of PBS and a 250 μL-aliquot was centrifuged at 20,800g for 40 min and the pellet frozen at – 80°C to prepare vesicle-derived proteins for further characterization. The 750 μL-aliquot of the suspension was centrifuged as above and the pellet resuspended in 200 μL of PBS and frozen at –80°C for nanoparticle tracking analysis, analytical gel-filtration and Vero cell translation and viability assays. In the case of donors 5–12, the whole suspension (1 mL) was centrifuged as above and the final pellet resuspended in 100 μL of PBS and frozen at −80°C for viability assays and mice experiments. The size and relative abundance of the isolated EVs were determined by nanoparticle tracking analysis (NTA) using the NanoSight RS300 system (Malvern Panalytical, Malvern, United Kingdom). Each sample was diluted (1:1000) in 0.1 μm filtered PBS. Three videos of 60s each were recorded and analyzed, calculating an average number of dimension and concentration (particles/mL). All samples were characterized with NTA 3.4 analytical software.

#### Protein extraction from EVs

Each pellet of EVs obtained by the 250 μL-aliquot (see above) was lysed by adding 25 μL of RIPA buffer containing a protease inhibitor cocktail (cOmplete, Roche, Sigma-Aldrich, USA) and incubated for 10 min with occasional stirring. Then, each sample was centrifuged at 14,000g for 10 min at 4°C to discard membranes and the supernatant recovered and frozen at –80°C. Vesicle-derived proteins were quantified by Bradford assay, with BSA as standard.

#### Characterization of blood cell-derived EVs by WES

EVs (10,400g-20,800g) were characterized by WES (quantitative capillary Western Blot) performed on total proteins extracted from vesicles. Each sample was prepared according to the manufacturer’s instructions by diluting proteins with a provided sample diluent and a master mix containing DTT and incubated 5 min at 95°C. Then, the marker, the sample, the antibody diluent for the blocking step, the primary antibody, the secondary antibody, streptavidin-HRP and Luminol-Peroxide mix were added to each well of the plate. The plate and the capillaries were used to start the automated Western Blot analysis and the quantitative immunodetection (Wes, ProteinSimple, Bio-techne).

#### Analytical gel-filtration of EVs

A representative preparation of EVs (control vesicles, Stx2a vesicles and Stx2a+NAB815 vesicles) was analyzed by gel-filtration column chromatography (Sephacryl S-500 HR, 0.5 cm × 28 cm, 5.5 mL-volume, 200 nm exclusion size). After equilibration with 20 mM Tris-HCl (pH 7.4) containing 150 mM NaCl, 100 μL-aliquots of each sample were separately loaded on the column. Then 5 mL of the buffer were added and 250 μL-fractions were collected. The absorbance at 280 nm was recorded and Vero cell protein synthesis assay was used to detect Stx2a in the fractions.

#### Histopathology

Histopathologic analyses were performed on deceased mice (first model) or at the conclusion of the experiments (second model) following euthanasia via gaseous overdose of isoflurane (Iso–Vet, Piramal Critical Care, Voorschoten, The Netherlands), followed by cervical dislocation.

Kidney tissue was fixed in 10% neutral formaldehyde for 2 days, and sections were stained with hematoxylin and eosin and periodic acid-Schiff (PAS). PAS was used to identify proximal tubules, which have a thick brush border. Slides were read by a veterinary pathologist (Dipl. ECVP) who was blind to the study group identifications. Damage of the tubules was considered as attenuation of tubular epithelium with loss of brush border and blebbing of apical cytoplasm with increased eosinophilic staining, tubular epithelial nuclei with condensed chromatin, increased basophilic staining or loss of distinct nuclear contour, tubular lumens filled with sloughed-off necrotic tubular epithelial cells, fibrin debris or hyaline casts, or hydropic degeneration of epithelial cells. All these lesions were considered acute tubular injuries (ATIs).

PAS-stained kidney sections were evaluated semiquantitatively under a light microscope with a 4× objective by assessing the presence of ATIs as follows: score 0 (minimal): <10% of parenchyma; score 1 (mild): 11–25%; score 2 (moderate): 26–50%; score 3 (marked): 51–75%; score 4 (severe): >75%. Upon identification of highly damaged areas, the numbers of detached cells present in a 10 × 10 mm grid area were counted using a 1-cm^2^ 10 × 10 grid reticle at 400× magnification (total area 1 mm^2^). The same method was used for ATI counting (all the cells with different types of damage) in a smaller area (0.1875 mm^2^).

### Quantification and statistical analysis

Statistical analysis was performed with GraphPad Prism 8 software. The number of replicates or of subjects (n), the type of statistical test, and the statistical significance are indicated in the text or in legends to figures. Differences in continuous variables (means, SD) were tested with a t-test after controlling the normality of their distribution. Correlation between variables was assessed by the Pearson correlation coefficient. Significance, for two-group comparisons, was calculated by two-tailed unpaired Mann-Whitney t-test or two-tailed unpaired t-test. Significance, for comparison between multiple groups, was calculated by one-way ANOVA with Tukey’s multiple comparison test or Dunnett’s multiple comparison test. Comparison of survival curves obtained in animal experiments was performed by Log-rank (Mantel-Cox) test. A *p* value < 0.05 was considered statistically significant.
