# Supplementary material for: An antibiotic derivative as a new potential tool in the prevention of hemolytic uremic syndrome
Source: iScience. 2025 Jul 7;28(8):113076. doi: 10.1016/j.isci.2025.113076 (PMC12309968; doi:10.1016/j.isci.2025.113076)
Supplement: Document S1. Figures S1–S7 [file mmc1.pdf]

iScience, Volume *28*

Supplemental information

An antibiotic derivative as a new potential tool in the prevention of hemolytic uremic syndrome

Elisa Varrone, Luciano Consagra, Domenica Carnicelli, Elisabetta Galassi, Beatrice Munari, Elisa Porcellini, Marta Pluchino, Giorgia Rossi, Federico Parenti, Catia Barboni, Barbara Brunetti, Francesca Ricci, Pier Luigi Tazzari, Francesco Manoli, Ilse Manet, Paola Paterini, Gianluca Storci, Massimiliano Bonafè, Alejandro Hochkoeppler, Anna Zaghini, Stefano Morabito, Gianluigi Ardissino, Timo Vaara, Martti Vaara, and Maurizio Brigotti

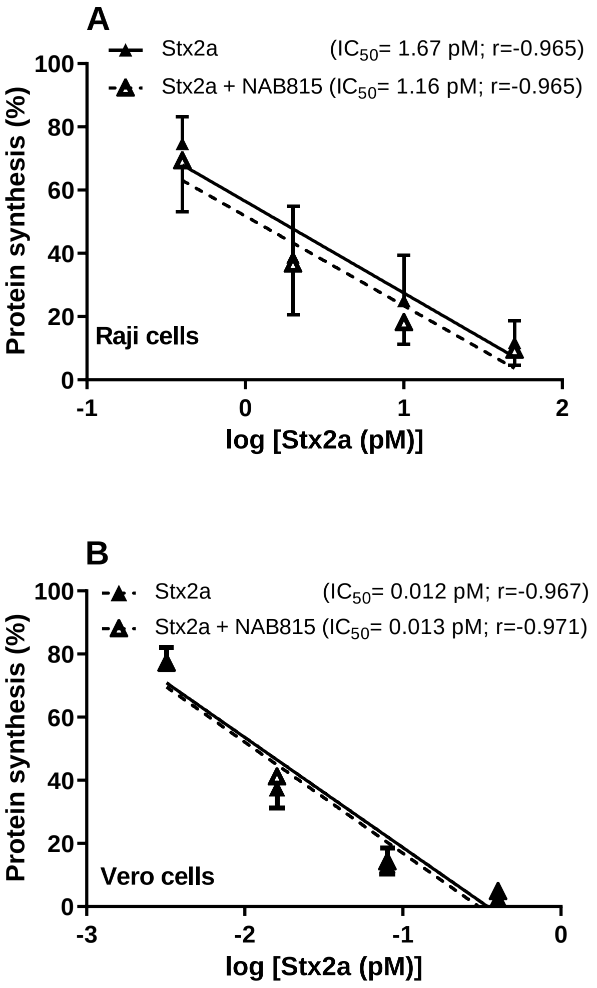

Figure S1. Effect of NAB815 on the inhibition of translation induced in Raji and Vero cells by Stx2a, related to Figure 1.

(A-B) The IC_{50} of Stx2a in impairing Raji cells' (A) or Vero cells' (B) translation in the absence (continuous line) and in the presence of 0.3 µg/ml NAB815 (dotted line) were calculated by the linear regression between the mean percentage of translation obtained in two experiments and the log of Stx2a concentrations. Error bars represent SD. Pearson correlation coefficient (r) was used to assess the correlation between variables.

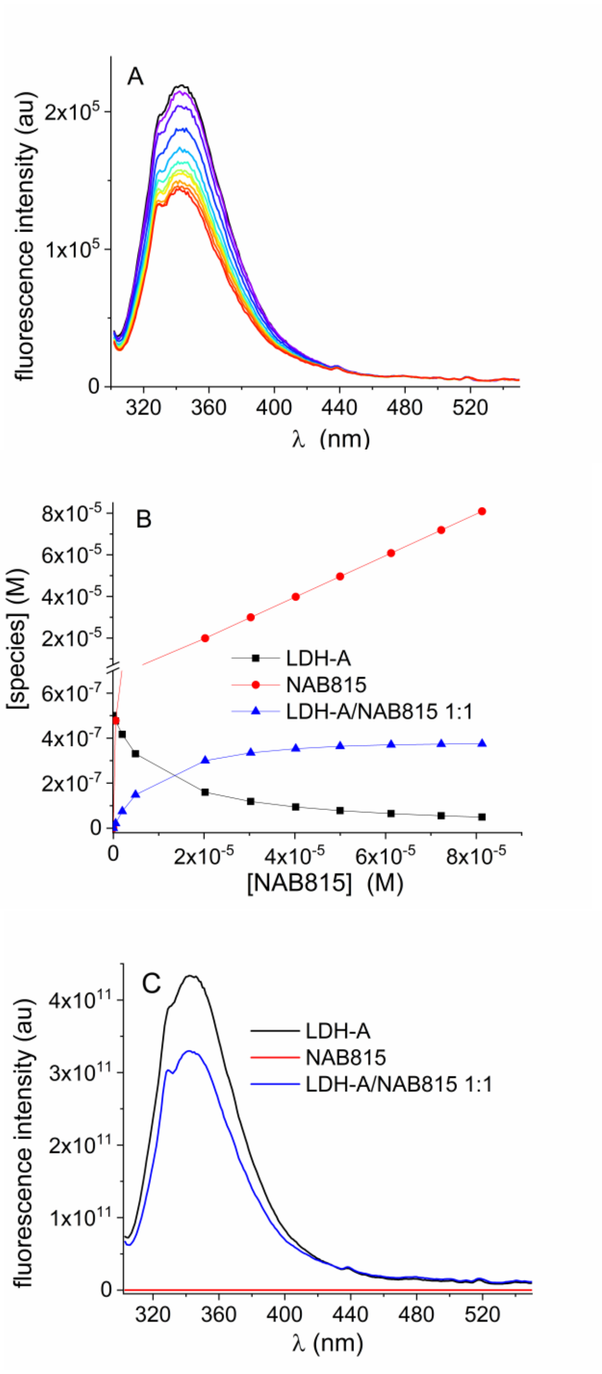

Figure S2. Determination of the binding of NAB815 to LDH-A, related to Figure 2.
LDH-A (0.5 μM in 500 μl of PBS) was excited at 295 nm to collect the fluorescence spectrum with maximum at 349 nm. Subsequently, increasing amounts of NAB815 were added to the final concentrations of 0.5-80 μM and the fluorescence was measured after each addition (volume of the sample at the end of the assay, 605 μl). The results were corrected at each experimental point for the dilution of the protein due to the addition of the antibiotic. NAB815 excited at the same wavelength is not fluorescent.
(A) Fluorescence spectra of the titration of LDH-A (black line) with increasing amounts of NAB815 (coloured lines).
(B) Plot of the equilibrium concentrations of the various species (LDH-A, NAB815 and the 1:1 complex) *vs* the total concentration of NAB815.
(C) Calculated fluorescence spectra of LDH-A as free species and complexed with NAB815.

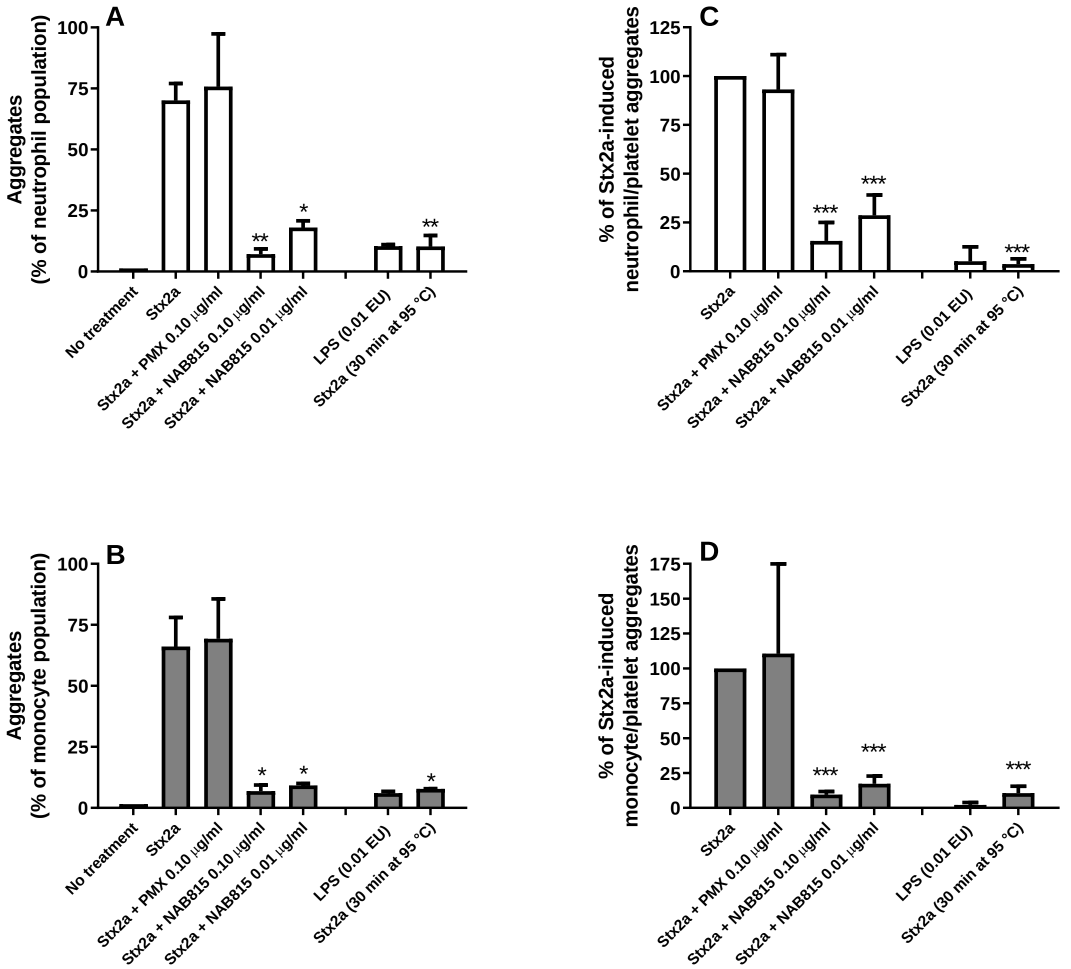

Figure S3. Effects of NAB815 on the formation of neutrophil/platelet or monocyte/platelet aggregates induced by Stx2a, related to Figure 1.

Non-fractionated blood samples (1 ml) from healthy donors were incubated for 4 h at 37 °C with Stx2a (1 nM) in the presence or in the absence of different concentrations of polymyxin B (PMX) or NAB815. Then, the formation of neutrophil/platelet or monocyte/platelet aggregates was assessed by direct flow cytometric analysis as described in Methods.

(A-B) Values obtained in a representative experiment performed in duplicate with blood from a single donor are expressed as percentage of aggregates on the neutrophil (A) or monocyte (B) populations, a control with the parent antibiotic polymyxin B (PMX) was also added; data are means ± SD (n=2).

(C-D) Values obtained with different human donors (n=3) are expressed as percentage of total aggregates formed by Stx2a; a control with the parent antibiotic polymyxin B (PMX) was also added; data are means ± SD. After incubation of human blood with Stx2a, in the absence of antibiotics, the percentages of neutrophil/platelet aggregates and of monocyte/platelet aggregates on the whole population of neutrophils or monocytes were 67.8 ± 17.2% and 62.5 ± 10.7% (mean ± SD, n=3), respectively.

Controls for contaminating LPS or with heat-inactivated Stx2a are also shown in the panels. *$p<0.05$, **$p<0.01$, ***$p<0.001$ (two-tailed unpaired t-test).

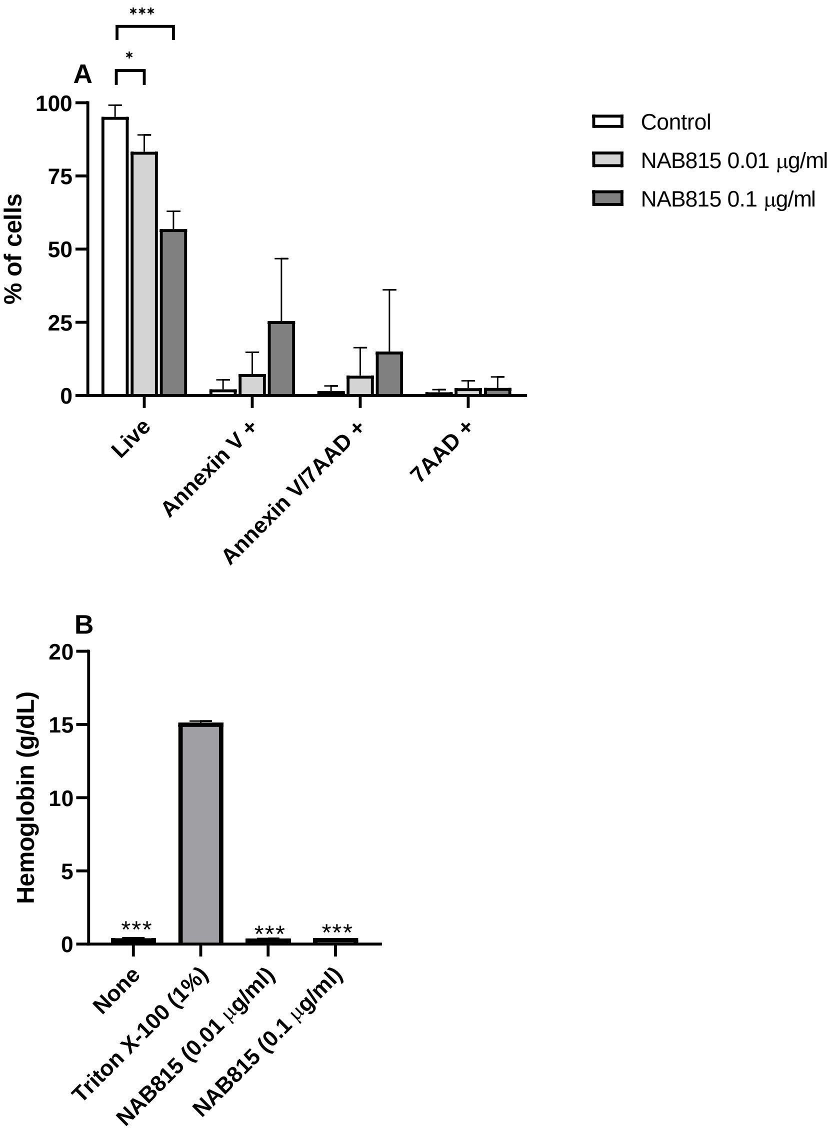

Figure S4. Effect of NAB815 on human white and red blood cells, related to Figure 1.
(A) Leukocytes isolated from 1 ml blood sample from healthy donors (n=3) were incubated for 4 h at 37 °C with NAB815 and analysed by flow cytometry after staining with annexin V and 7-AAD. Annexin V+ cells are early apoptotic cells, annexin V/7-AAD+ cells are late apoptotic cells, 7-AAD+ cells are necrotic cells; *p<0.05; ***p<0.001 (two-tailed unpaired t-test).
(B) Red cells isolated from a healthy donor were incubated with the indicated concentrations of NAB815 or triton X-100 as positive control. After 4 h at 37 °C the concentration of free haemoglobin present in the supernatant was determined by the absorbance at 540 nm according to the extinction molar coefficient (A_{540} = 15,300 M^{-1}). The experiment was performed in duplicate. ***p<0.0001 *vs* triton X-100 (two-tailed unpaired t-test).

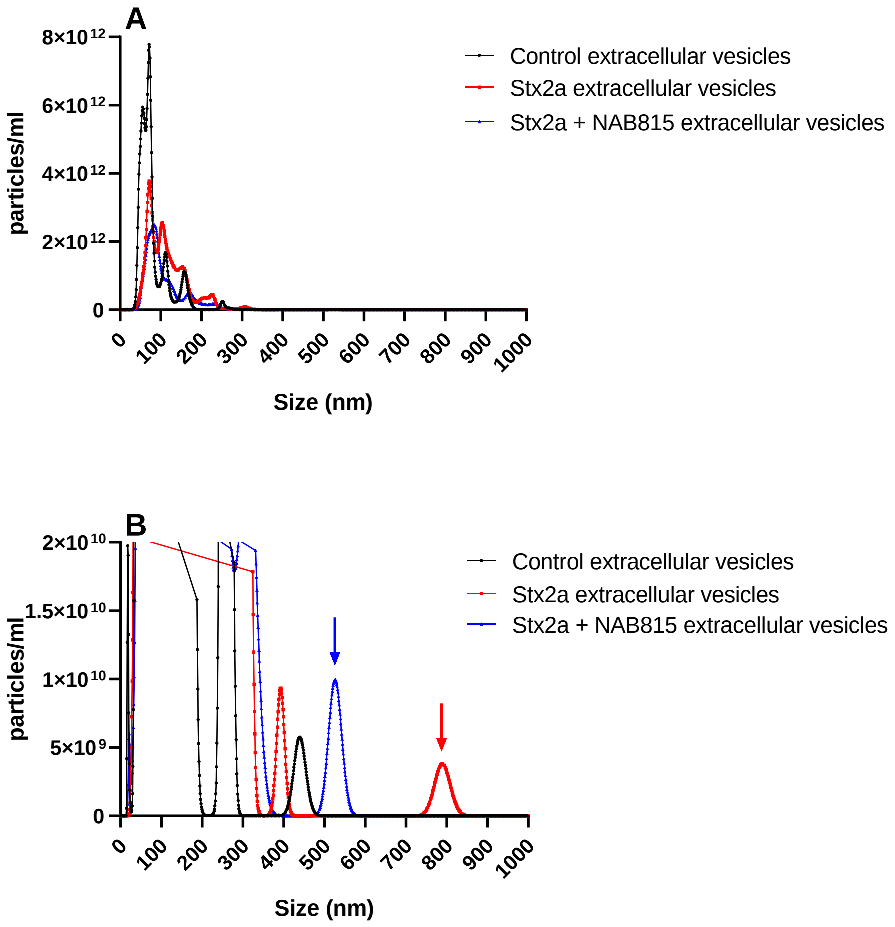

Figure S5. Determination of the number and size of EVs by nanoparticle tracking analysis, related to Figure 3. (A-B) Nanoparticle tracking analysis of EVs obtained after treatment of human blood from a representative donor with 2 nM Stx2a in the absence (red line) or in the presence (blue line) of 0.01 µg/ml NAB815, or vehicle (black line). The vesicles were isolated by differential centrifugation as described in Methods, 10^6-fold diluted and applied to NanoSight. Data are expressed as number of particles/ml of the different populations of EVs resolved by the technique and differing each other in diameter by 0.5 nm. Red and blue arrows indicate the larger vesicle components. The same data are reported in panels A and B which differs only in the scale of the y-axis.

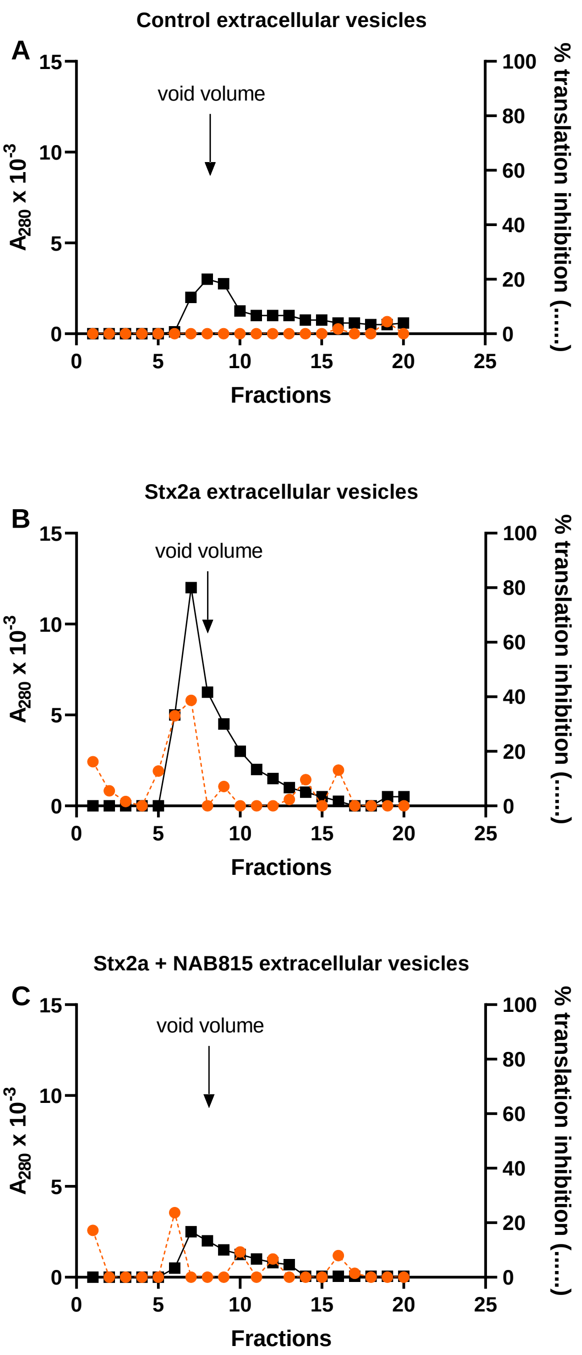

Figure S6. Gel-filtration analysis of human EVs, related to Figure 3.
(A-C) Gel-filtration analysis of the populations of EVs isolated after treatment of human blood from a representative donor with PBS (A) or with 2 nM Stx2a in the absence (B) or in the presence (C) of 0.01 µg/ml NAB815. Aliquots of the preparations were applied to a Sephacryl S-500 HR column (exclusion size of 200 nm) as described in Methods. Black arrows indicate the limit of the void volume containing the excluded particles, black squares (continuous lines) represent the absorbance at 280 nm of each fraction and orange circles (dotted lines) the percentage of inhibition of protein synthesis obtained by adding aliquots of the fractions to Vero cell translation assay performed as described in Methods.

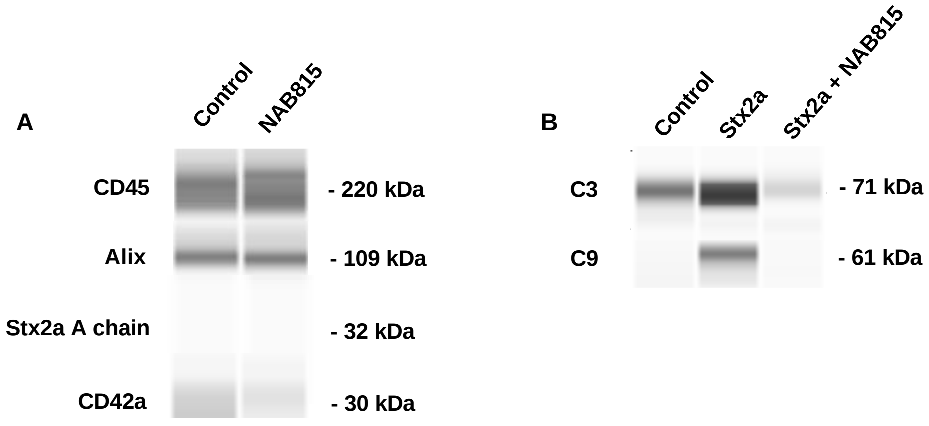

Figure S7. Quantitative capillary Western Blot (WES) analysis of the proteins derived from human EVs, related to Figure 4.

(A) Human EVs were isolated after treatment of human blood with vehicle or with 0.01 µg/ml NAB815 and their proteins extracted as described in Methods. A representative WES of the analysed antigens (Alix, CD45, CD42a) and associated proteins (Stx2a) is shown. Different primary antibodies were used: rabbit polyclonal anti-Alix 1:50 (Novus Biological) as EV marker; mouse monoclonal anti-CD45 1:250 (BD Transduction Laboratories) as leukocyte marker; rabbit polyclonal anti-CD42a 1:10 (GeneTex) as platelet marker; rabbit polyclonal anti-Stx2a 1:50 (Dr. Stefano Morabito, ISS Rome) to detect the toxin.

(B) Human EVs were isolated after treatment of human blood with vehicle or with 2 nM Stx2a in the absence or in the presence of 0.01 µg/ml NAB815 and their proteins extracted as described in Methods. A representative WES of the analysed antigens; i.e. complement factor 3 (C3) and complement factor 9 (C9) is shown. Different primary antibodies were used: mouse monoclonal anti-C3 1:10 (Hycult Biotech) and mouse monoclonal anti-C9 1:10 (Hycult Biotech) to detect complement factors.
